# Endovascular Treatment Modalities for Infrapopliteal Artery Disease: A Bayesian Network Meta-Analysis with Exploratory Evaluation of Retrievable Scaffold Therapy

**DOI:** 10.1007/s00270-026-04452-0

**Published:** 2026-04-30

**Authors:** Muhammad Saleem, Muhammad Haider Tariq, Muhammad Mudassar, Ajit Brar, Aleena Usman, Arsalan Jibbran, Fatima Tariq, Anwar Zaitoun

**Affiliations:** 1 Michigan State University, East Lansing, MI USA; 2 Northwest Medical Center, Tucson, AZ USA; 3 King Edward Medical University, Lahore, Pakistan; 4 Hurley Medical Center, Flint, MI USA; 5 Faisalabad Medical University, Faisalabad, Pakistan

**Keywords:** Atherectomy, Infrapopliteal artery, Meta-analysis

## Abstract

**Background:**

Endovascular options for infrapopliteal artery disease include plain balloon angioplasty (PTA), drug‑coated balloons (DCB), drug‑eluting and bare‑metal stents (DES), and atherectomy. Retrievable scaffold therapy (RST) has recently emerged as a temporary scaffolding strategy used with DCB, but its comparative effectiveness remains uncertain.

**Methods:**

A comprehensive literature search identified 21 randomized and 4 single‑arm trials (*n* = 3184). Eligible studies reported at least one prespecified outcome: 30‑day major adverse events (MAE), 12‑month all‑cause mortality, 6‑month clinically driven target lesion revascularization (CD‑TLR), or 6‑month major amputation. Random‑effects models generated odds ratios (ORs) with 95% credible intervals (CrIs). SUCRA values summarized treatment rankings.

**Results:**

Atherectomy ranked best for 30‑day MAE (SUCRA 77.2%), though no treatment yielded a statistically significant reduction in the Odds Ratio. It was also associated with a significantly reduced 12‑month mortality versus PTA (OR 0.39, 95% CrI 0.15–0.90; SUCRA 97.8%). For 6‑month CD‑TLR, atherectomy (OR 0.26, 95% CrI 0.00–0.62), DCB (OR 0.42, 95% CrI 0.30–0.58), and DES (OR 0.43, 95% CrI 0.19–0.88) showed significant benefit. No treatment significantly reduced major amputation. RST showed favorable but nonsignificant reductions, indicating a smaller number of available studies, consistently ranking mid‑tier across all outcomes. Meta‑regression identified CKD and longer lesion length as predictors of higher CD‑TLR risk.

**Conclusions:**

Atherectomy showed the most consistent benefits across mortality and reintervention outcomes, while RST demonstrated promising but inconclusive performance. Larger randomized trials are needed to clarify RST’s role in infrapopliteal revascularization.

**Graphic Abstract:**

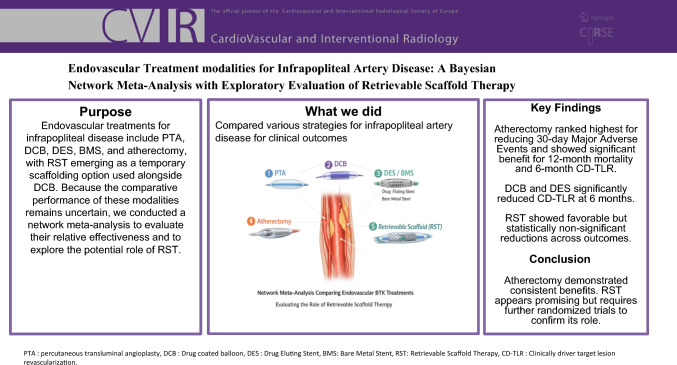

**Supplementary Information:**

The online version contains supplementary material available at 10.1007/s00270-026-04452-0.

## Introduction

Peripheral artery disease (PAD) affects more than 200 million individuals worldwide [1] and remains a major cause of morbidity, mortality, and limb loss [2]. Among its clinical manifestations, chronic limb‑threatening ischemia (CLTI) represents the most advanced form, affecting an estimated 11% of patients with PAD [3]. Patients with CLTI typically present with ischemic rest pain, non‑healing ulcers, or gangrene, and face poor outcomes, including amputation without timely revascularization [4]. Mortality rates for CLTI are high, with a three-year mortality rate approaching 40% [5, 6], which underscores the systemic and progressive nature of the disease.

Revascularization of infrapopliteal arteries is a Class I guideline‑recommended therapy for improving perfusion, promoting wound healing, and reducing the risk of amputation in patients with CLTI [7, 8]. Recently, endovascular therapy has become increasingly popular, as it offers a minimally invasive approach with outcomes similar to bypass surgery [9]. However, despite its central role, infrapopliteal endovascular intervention remains a technically challenging procedure. Below‑the‑knee (BTK) arteries are characterized by small vessel caliber, diffuse and long‑segment disease, heavy calcification, impaired distal runoff, and a high propensity for elastic recoil and dissection [10]. Percutaneous transluminal angioplasty (PTA) with a plain balloon has served as the foundational therapy for decades, yet its durability is limited by high restenosis and reintervention rates [11, 12]. To address these limitations, multiple device‑based strategies have emerged, including drug‑coated balloons (DCBs), drug‑eluting stents (DES), bare‑metal stents (BMS), atherectomy‑assisted angioplasty, bioresorbable scaffolds, and dissection repair systems. However, evidence supporting these modalities remains heterogeneous, and prior comparative studies have yielded inconsistent results [13–15], leaving uncertainty regarding the optimal endovascular approach for BTK disease.

Drug‑coated technologies, in particular, have garnered substantial interest due to their potential to inhibit neointimal hyperplasia, a key driver of restenosis. While paclitaxel‑coated devices have historically dominated the peripheral space, concerns regarding long‑term safety have stimulated interest in limus‑based alternatives [16]. Yet effective arterial delivery of limus-based drugs has been challenging in the absence of a permanent stent platform [17]. Retrievable scaffold therapy (RST), incorporating a temporary spur‑based scaffold to facilitate drug transfer while avoiding a permanent implant, represents a novel approach designed to overcome these limitations. Because RST is an emerging technology supported primarily by single‑arm studies, its comparative performance remains uncertain and warrants exploratory evaluation.

Given the expanding landscape of BTK endovascular therapies and the lack of definitive comparative evidence, a comprehensive synthesis of available data is needed to guide clinical decision‑making and inform future research. Therefore, we conducted a systematic review and network meta-analysis to compare the safety and efficacy of contemporary endovascular modalities for infrapopliteal artery disease, including PTA, DCB angioplasty, DES, BMS, atherectomy‑assisted angioplasty, and RST. Recognizing the limited evidence base for RST, we prespecified an exploratory analysis to contextualize its early outcomes relative to established therapies. Our goal was to provide an integrated, evidence‑based assessment of treatment strategies, leveraging both direct and indirect comparisons to clarify the current therapeutic landscape for BTK revascularization.

## Methodology

This Study-level Bayesian network meta-analysis was conducted in accordance with the Preferred Reporting Items for Systematic Reviews and Meta-Analyses (PRISMA) 2020 guidelines [18] and was prospectively registered with PROSPERO (Registration ID: CRD420251235114). A comprehensive search of MEDLINE, Embase, the Cochrane Central Register of Controlled Trials (CENTRAL) via Ovid, and ClinicalTrials.gov was performed from inception till November 10, 2025. Search terms incorporated both Medical Subject Headings (MeSH) and keywords related to infrapopliteal arterial disease, tibial artery revascularization, percutaneous transluminal angioplasty, drug-coated balloons, drug-eluting stents, bare-metal stents, atherectomy, and retrievable scaffold therapy. No date restrictions were applied, and bibliographies of eligible studies and relevant reviews were screened to identify additional publications. Only English language studies were included. The full search strategy is uploaded to PROSPERO.

Eligible studies were clinical trials enrolling adults with infrapopliteal arterial disease undergoing endovascular revascularization with one of the pre-specified modalities of intervention. Studies were required to report arm-level data for at least one of the prespecified outcomes: the primary outcome of Major Adverse Events (MAE) at 30 days, defined as a composite of all-cause mortality, major amputation, and any re-intervention of the target limb, and the secondary outcomes of all-cause mortality at 12 months, clinically-driven target lesion revascularization (CD-TLR) at 6 months, or major limb amputation at 6 months. All comparator categories of interest were prespecified: percutaneous transluminal angioplasty (PTA), Angioplasty with drug-coated balloon (DCB), Balloon angioplasty followed by drug-eluting stent (DES), Balloon angioplasty followed by bare-metal stent (BMS), Balloon angioplasty followed by atherectomy, and Balloon angioplasty combined with retrievable scaffold therapy (RST). Because available RST studies were limited to single-arm designs, these were retained if they reported extractable binomial outcome data; the Bayesian arm-based modeling framework permitted their inclusion by allowing single-arm evidence to contribute to baseline risk estimation.

**Fig. 1:**
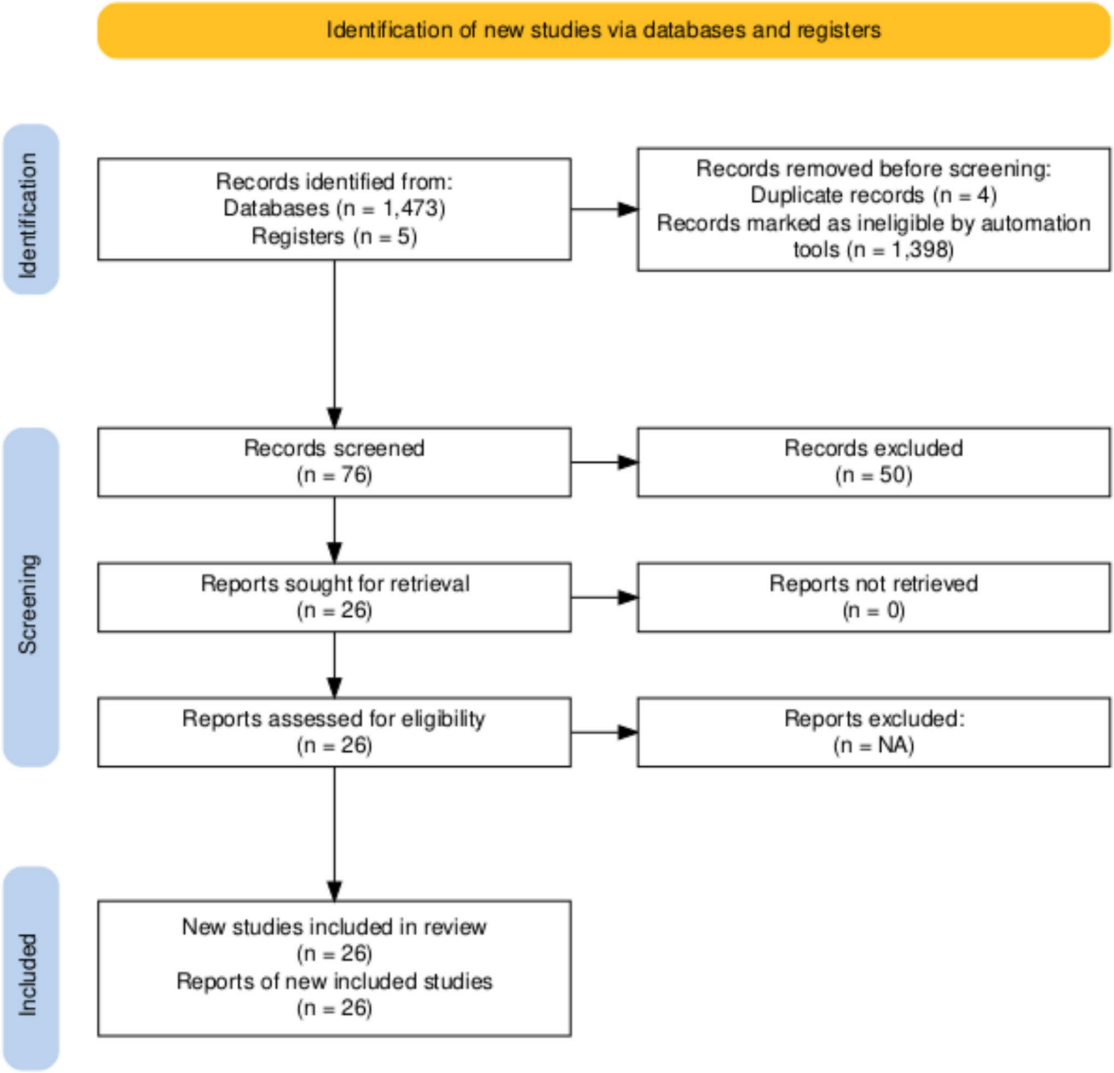
PRISMA flow diagram illustrating the study selection process. The diagram was generated using an R package and a Shiny application [19]

Studies were excluded if they enrolled patients with mixed lesion locations without separate infrapopliteal data, lacked extractable event counts or sample sizes, or did not report any primary or secondary outcomes. Two reviewers independently screened all titles and abstracts, followed by a full-text review. Discrepancies were resolved by consensus or by consultation with a third reviewer. The full study-selection process is demonstrated in the PRISMA [18] flow diagram below (Fig. 1):

Data extraction was performed in duplicate using a standardized Excel sheet capturing study characteristics, patient demographics, lesion length, comorbidities, treatment assignment, and event counts. Extracted covariates included mean age, prevalence of diabetes mellitus, smoking prevalence, prevalence of chronic kidney disease, and lesion length. Risk of bias was evaluated independently by two reviewers using the Cochrane Risk of Bias 2.0 tool [20], assessing randomization processes, deviations from intended interventions, missing outcome data, outcome measurement, and selective reporting. For non-randomized studies evaluating RST, the ROBINS-I tool [21] was used.

A Bayesian arm-based network meta-analysis was conducted using JAGS through R. Each study arm was modeled using a binomial likelihood with a logit link, decomposing the arm-level log-odds into a study-specific baseline effect and a treatment-specific relative effect. Percutaneous transluminal angioplasty served as the reference treatment. The arm-based approach was chosen because it preserves within-study correlations, allows incorporation of multi-arm trials without data duplication, and permits inclusion of single-arm studies by informing the posterior distribution of study-specific baseline risks. Random effects were applied to account for between-study heterogeneity, and weakly informative priors were assigned to variance parameters. Convergence was confirmed by trace plots and the Gelman–Rubin statistic.

Odds ratios with 95% credible intervals were derived from posterior samples for all pairwise treatment comparisons. Network geometry was visualized with node-link diagrams, with **node sizes reflecting the number of contributing studies** and edges weighted by the number of direct comparisons. Treatment rankings were summarized using Surface Under the Cumulative Ranking Curve (SUCRA) values derived from posterior rank distributions. To evaluate publication bias, a study-level funnel plot was generated using logit-transformed event rates plotted against their standard errors. **An Egger regression test adapted for arm-based data was performed to assess relative funnel asymmetry. Sensitivity analyses were conducted by repeating the network meta-analysis after excluding studies identified as high-risk for publication bias based on significant asymmetry patterns.**

Study-level Bayesian meta-regression analyses were performed for each prespecified covariate (mean age, diabetes mellitus prevalence, smoking prevalence, chronic kidney disease prevalence, and lesion length) individually, using the same arm-based model structure with an added covariate term acting on the relative treatment effect. All analyses were conducted using R version 4.5.1.

## Results

### Study Characteristics and Assumption of Transitivity

Our analysis included 25 studies enrolling 3184 participants. Patients were predominantly elderly (mean age 70–75 years) with a high prevalence of diabetes (typically > 70%, and 100% in several studies). Smoking prevalence varied widely (6–80%), while chronic kidney disease was inconsistently reported and ranged from 8 to 45%. Lesion lengths differed substantially, with some trials enrolling short focal lesions (15–30 mm), whereas other studies treated longer, diffuse disease (100–190 mm). While age and diabetes prevalence were broadly consistent across studies, heterogeneity in lesion length and comorbidity reporting may represent potential effect modifiers, warranting cautious interpretation of transitivity. Detailed study characteristics are presented in Table 1. A meta-regression was performed to explore the effect of these covariates on outcomes of interest (Table 2).

**Table 1:** Baseline characteristics of included clinical studies with a total of 3184 participants

Study	Treatment	participants	Mean Age	Diabetics (%)	smokers (%)	CKD (%)	lesion length (mm)	Rutherford Class 3 (%)	Rutherford Class 4 (%)	Rutherford Class 5 (%)	Rutherford Class 6 (%)	Calcium
Moderate or severe (% lesions)												
SINGA-PACLI [22]	DCB	70	61	94	33		90.3	0	3	81	16	70
	PTA	68	64	94	38		81.8	0	4	62	32	60
Haddad [23]	DCB	48	52—74	98	67					100		
	PTA	45	53—77	93	80					100		
AcoArtII-BTK [24]	DCB	61	70.7	74			169.9	2	44	39	15	46.1
	PTA	59	70.8	71			179.9	0	41	47	12	40.9
AcoArt-BTK [25]	DCB	52	75.4	100		40	168	0	7.2	83.5	9.3	61.3
	PTA	53	74.8	93		45	187	0	7.2	85.5	7.3	66.6
DebateBTK [26]	DCB	65	74	100	20		129	0	2.8	78.9	18.3	25
	PTA	67	75	100	10.4		131	0	4.2	81.9	13.9	28.2
LutonixBTK [27]	DCB	287	72.9	71.1	59.2	23.7	111.8	9.1	34.8	56.1	0	15.1
	PTA	155	72.9	68.4	57.4	16.8	94.7	10.3	33.5	56.1	0	13.2
BIOLUXPII [28]	DCB	36	72.9	61.1	55.6	27.8	113.1	19.4	5.6	72.2	0	8.8
	PTA	36	69.6	72.2	55.6	27.8	115	13.9	5.6	72.2	0	2.6
INPACTDEEP [29]	DCB	239	73.3	75.7	51.9	8.6	102	0	14.2	84.1	1.7	13.7
	PTA	119	71.7	68.9	49.6	12.5	129	0.8	17.6	77.3	4.2	10.5
IDEAS [30]	DCB	25	67.6	76	36	44	148		4.5(3,5)b			44
	DES	25	75.3	64	24	32	127		4.5(4,5)b			50
ACHILLES [31]	DES	99	72.4	64.6	38.4		26.9		4.1		0	15.1
	PTA	101	74.3	64.4	26.3		26.8		4.0		0	15.2
PADI [32]	DES	73	74.2	60.3	46.6	20.5	21.1	0	13.5	64.9	21.6	
	PTA	64	72.9	67.2	45.3	34.4	23.1	0	12.1	69.7	18.2	
SAVAL [33]	DES	130	73.3	63.8	71.5	17.7	68.1	0	50.8	49.2	0	57.1
	PTA	71	72.6	62	74.6	15.5	68.7	0	58.6	41.4	0	40.8
DESTINY [34]	DES	74	75	60	31	30	15.9		50	50	0	77
	BMS	66	76	50	33	33	18.9		39	61	0	74
Falkowski [35]	DES	25	68	40			17.4	64	24	12	0	
	BMS	25	70	40			18.2	72	16	12	0	
YUKONBTK [36]	DES	82	73.4	56.8		35.8	30	43.9	8.5	42.7	0	
	BMS	79	72.3	50.6		35.1	31	50.6	2.5	39.3	0	
Brodmann [37]	BMS	21	68.9	76.2			27.9	0		100		
	PTA	33	74.9	72.7			78.5	0		100		
InPertia [38]	BMS	24	72	66.7	58.3	20.8	24	0	16.7	80.3		
	PTA	27	72	70.3	63	11.1	24	0	29.7	70.3		
Randon [39]	BMS	16	72	62.5	6.2	12.5		0	25	75	0	
	PTA	22	72	54.5	18.2	36.4		0	4.5	95.5	0	
EXPAND [40]	BMS	45	72.5	71.1	62.2	42.2	34.1	35.6	4.4	60	0	
	PTA	47	73.3	66	53.2	40.4	29.5	34	4.3	59.6	0	
Rastan [41]	Atherectomy	40	71.5	70		60	191.6	30	10	55	0	70
	DCB	40	72.7	60		39	160.8	20	22.5	55	0	50.8
CALCIUM360 [42]	Atherectomy	25	70.7	72	25	25	91	0	48	44	8	93.1
	PTA	25	71.8	56	60	24	69	0	48	44	2	97.1
OPTIMIZEBTK [43]	Atherectomy	32	73.4	75	71.9	46.9	101.3	31.3	6.3	62.5	0	100
	DCB	34	76.5	58.8	50	11.8	78.8	23.5	11.8	64.7	0	88.3
DEEPER REVEAL [44]	RST	130	69.7									
DEEPERLIMUS [45]	RST	26	71	76.9	61.5	26.9	61	11.5	0	88.5		
DEEPER [46]	RST	22	69	95.5		36.4	135	18.2	13.6	68.2		100
DEEPEROUS [47]	RST	107	76	66		35	92.7					

Key variables include mean age, prevalence of diabetes, smoking, chronic kidney disease (CKD), and lesion length. Patients were predominantly elderly (mean age 70–75 years) with a high burden of diabetes (> 70% in most BTK studies, and 100% in several). Smoking prevalence ranged widely (6–80%), and CKD was inconsistently reported (8–45%). Lesion lengths varied substantially: DES/BMS trials typically enrolled short focal lesions (15–30 mm), whereas DCB and atherectomy studies treated longer, diffuse disease (100–190 mm)

**Table 2:** Meta‑regression analyses across outcomes. No covariates were significantly associated with 30‑day MAE, 12‑month mortality, or 6‑month major amputation

Column 1	Column 2	Column 3	Column 4
Covariate	Beta_Median	CI_Lower	CI_Upper
*MAE at 30 days*			
Mean age	− 0.4580783951	− 1.069108036	0.1917947636
Diabetes	− 0.08206799155	− 1.019982803	1.024474595
Smoker	− 0.590301692	− 1.777167569	0.5722192511
CKD	0.0517370328	− 1.202607062	0.9658497954
Lesion length	− 0.6953083678	− 1.709732331	0.2354573431
*Death at 12 months*			
Mean age	− 0.1012717245	− 0.3178685441	0.143603139
Diabetes	− 0.1380049718	− 0.4556246473	0.1344098438
Smoker	− 0.005880943597	− 0.3194320339	0.3621577618
CKD	0.07754685597	− 0.2077614705	0.3655025386
Lesion length	− 0.1604642058	− 0.4684330379	0.161624559
*CD-TLR at 6 months*			
Mean age	− 0.2963706529	− 0.6117594345	0.01060946223
Diabetes	− 0.2863741313	− 0.7605051418	0.1043872151
Smoker	0.02318662612	− 0.5001876996	0.4768791248
CKD	0.4450807827	0.04627050554	0.9021873198
Lesion length	0.4484038995	0.03881403179	0.9361920302
*Major amputation at 6 months*			
Mean age	− 0.2277165123	− 0.7975579082	0.4499664126
Diabetes	0.4809028507	− 0.4780611729	1.480985342
Smoker	− 0.7668943294	− 1.947475899	0.2947943268
CKD	− 0.1457120142	− 1.096617563	0.8525202272
Lesion length	− 0.5683678262	− 1.805707201	0.5052088846

In contrast, CKD (*β* = 0.45, 95% CI 0.05–0.90) and lesion length (*β* = 0.45, 95% CI 0.04–0.94) were independently associated with a higher risk of CD‑TLR at 6 months, while other patient characteristics did not consistently modify outcomes

### Primary outcome (Major Adverse Events at 30 days)

**Fig. 2:**
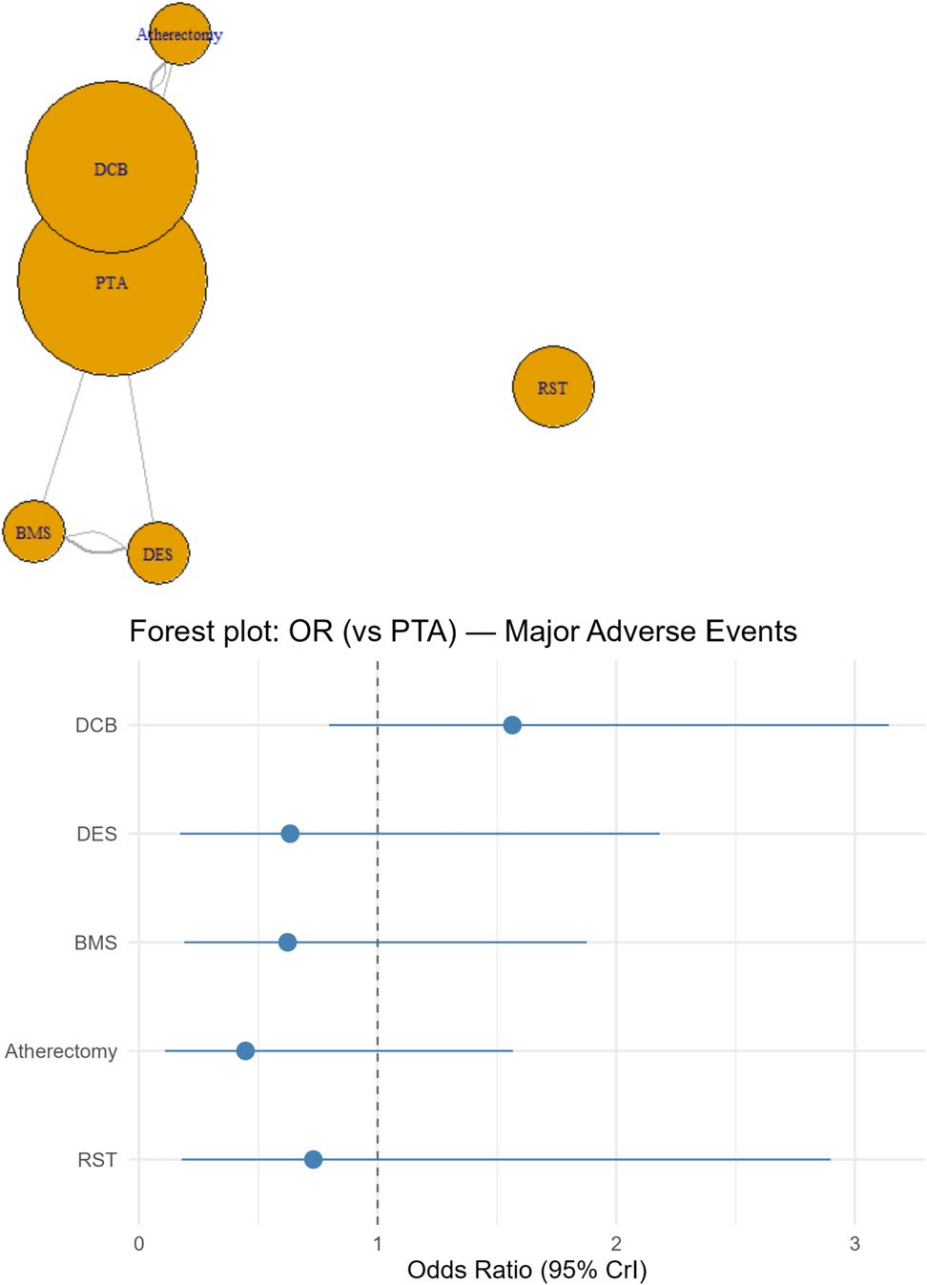
Network plot for the primary outcome (MAE at 30 days), which depicts a well-connected network except for RST, which only had single-arm studies, and a forest plot depicting odds ratios (ORs) of MAE at 30 days for the interventions of interest compared with PTA

Compared to PTA (Plain Balloon Angiolasty), DCB was associated with statistically insignificant higher odds of having the composite outcome of MAE at 30 days (OR of 1.56), while DES (OR: 0.63), BMS (OR: 0.62), Atherectomy (OR: 0.446), and RST (OR: 0.730) were associated with statistically insignificant lower odds of having the primary outcome (Fig. 2).

Treatment rankings (Supplementary Table 1A) revealed that Balloon angioplasty followed by Atherectomy had the highest probability of being ranked the best treatment per SUCRA (77.22%), followed by DES (61.38%), RST (53.6%), and Balloon angioplasty alone /PTA (35.5%), while DCB ranked the lowest (9.1%).

### Death at 12 Months

**Fig. 3:**
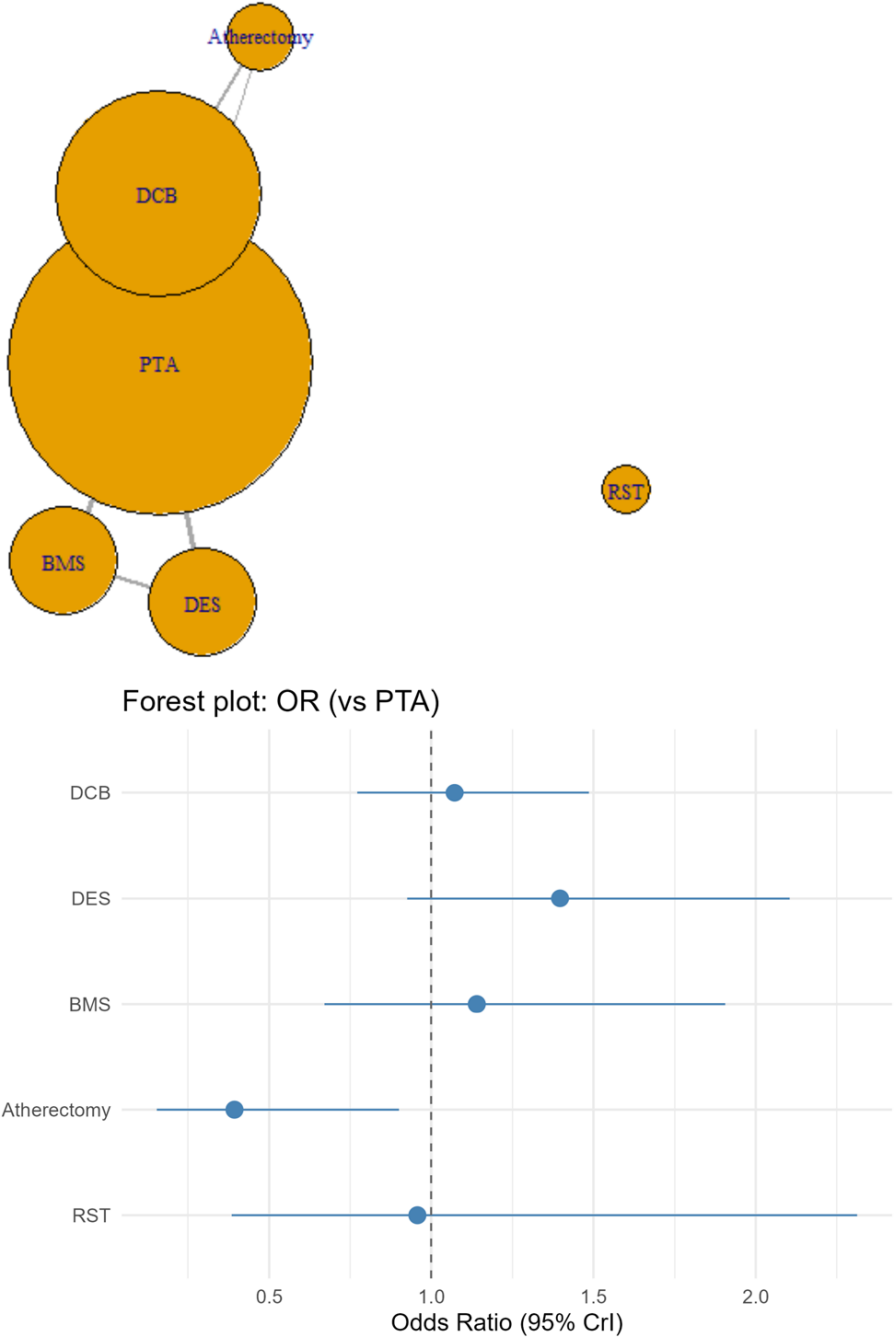
Network plot for the secondary outcome of death, which depicts a well-connected network except for RST, which only had single-arm studies, and a forest plot depicting odds ratios (ORs) of death for the interventions of interest compared with PTA

Compared to PTA (Fig. 3), Atherectomy was associated with statistically significant (OR: 0.39, 95% CrI 0.15, 0.90) odds, while RST was associated with statistically insignificant (OR: 0.96, 95% CrI 0.38, 2.31) lower odds of death at 12 months. DCB (OR: 1.07, 95% CrI 0.77, 1.48), DES (OR: 1.4, 95% CrI 0.93, 2.1), and BMS (OR: 1.14, 95% CrI 0.67, 1.9) were associated with statistically insignificant higher odds of mortality at 12 months.

Per SUCRA values (Supplementary Table 1 B), Atherectomy had the highest probability (97.8%) of being ranked the best treatment in terms of preventing mortality at 12 months, followed by PTA (55.2%), RST (52.6%), DCB (43.8%), BMS (37.7%), and DES (12.5%).

### Clinically Driven Target Lesion Revascularization (CD-TLR) at 6 Months

**Fig. 4:**
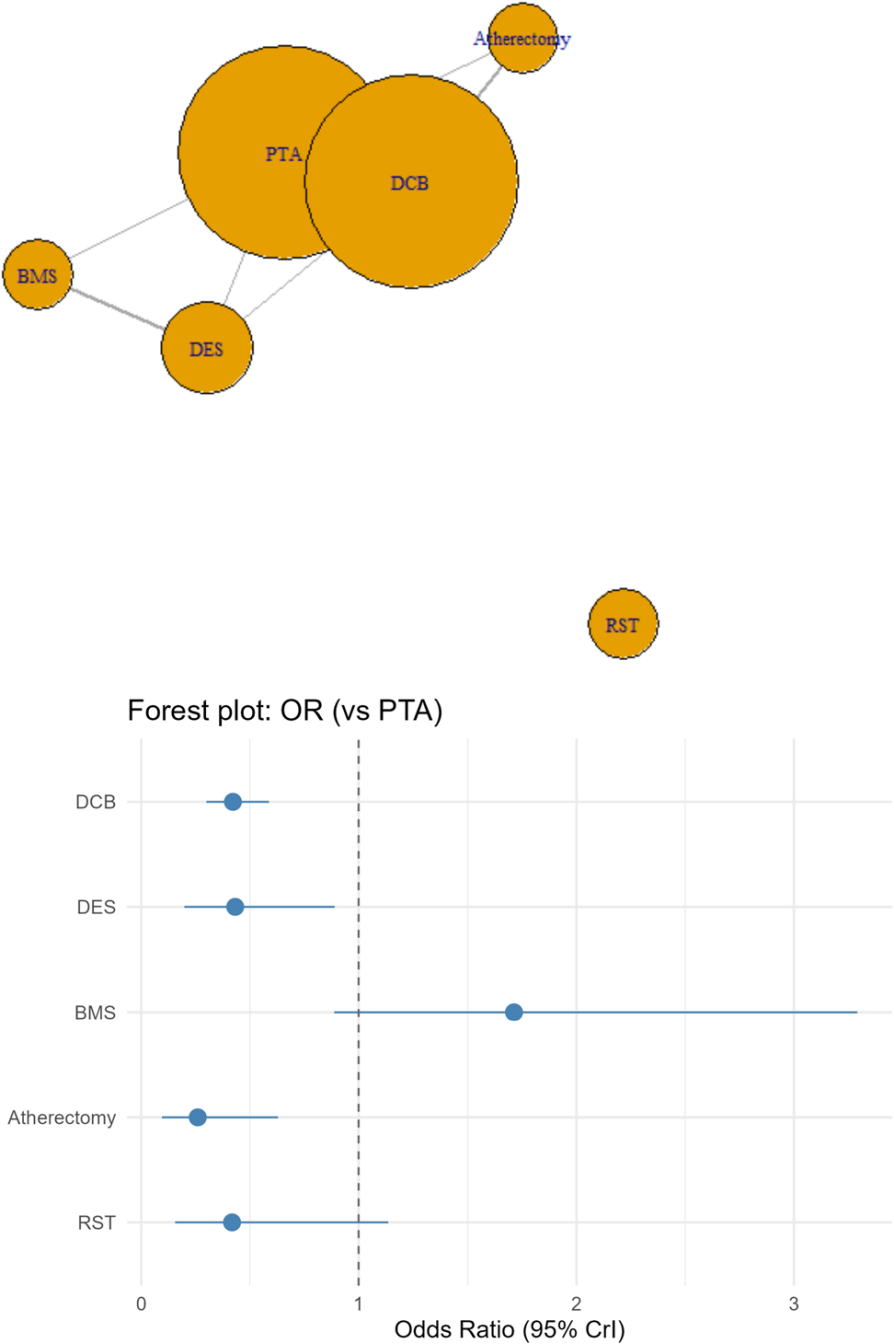
Network plot for the secondary outcome of CD-TLR at 6 months, which depicts a well-connected network except for RST, which only had single-arm studies, and a forest plot depicting odds ratios (ORs) of CD-TLR at 60 days for the interventions of interest compared with PTA

Compared with Balloon angioplasty alone (PTA), DCB (OR: 0.42, 5% CrI 0.3, 0.58), DES (OR: 0.43, 95% CrI 0.19, 0.88), and Atherectomy (OR: 0.26, 95% CrI 0.0, 0.62) were associated with lower odds of having CD-TLR at 6 months (Fig. 4). RST (OR: 0.42, 95% CrI 0.15, 1.13) showed statistically insignificant lower odds of CD-TLR, while BMS showed statistically insignificant higher odds (OR: 1.71, 95% CrI 0.88, 3.29) of CD-TLR at 6 months.

Balloon angioplasty followed by Atherectomy had the highest probability of being ranked the best treatment per SUCRA (88%), followed by RST (64%), DCB (63.2%), DES (62.7%), and Balloon angioplasty alone or PTA (20%), while BMS ranked the lowest (1.3%) (Supplementary Table 1C).

### Major Amputation at 6 Months

**Fig. 5:**
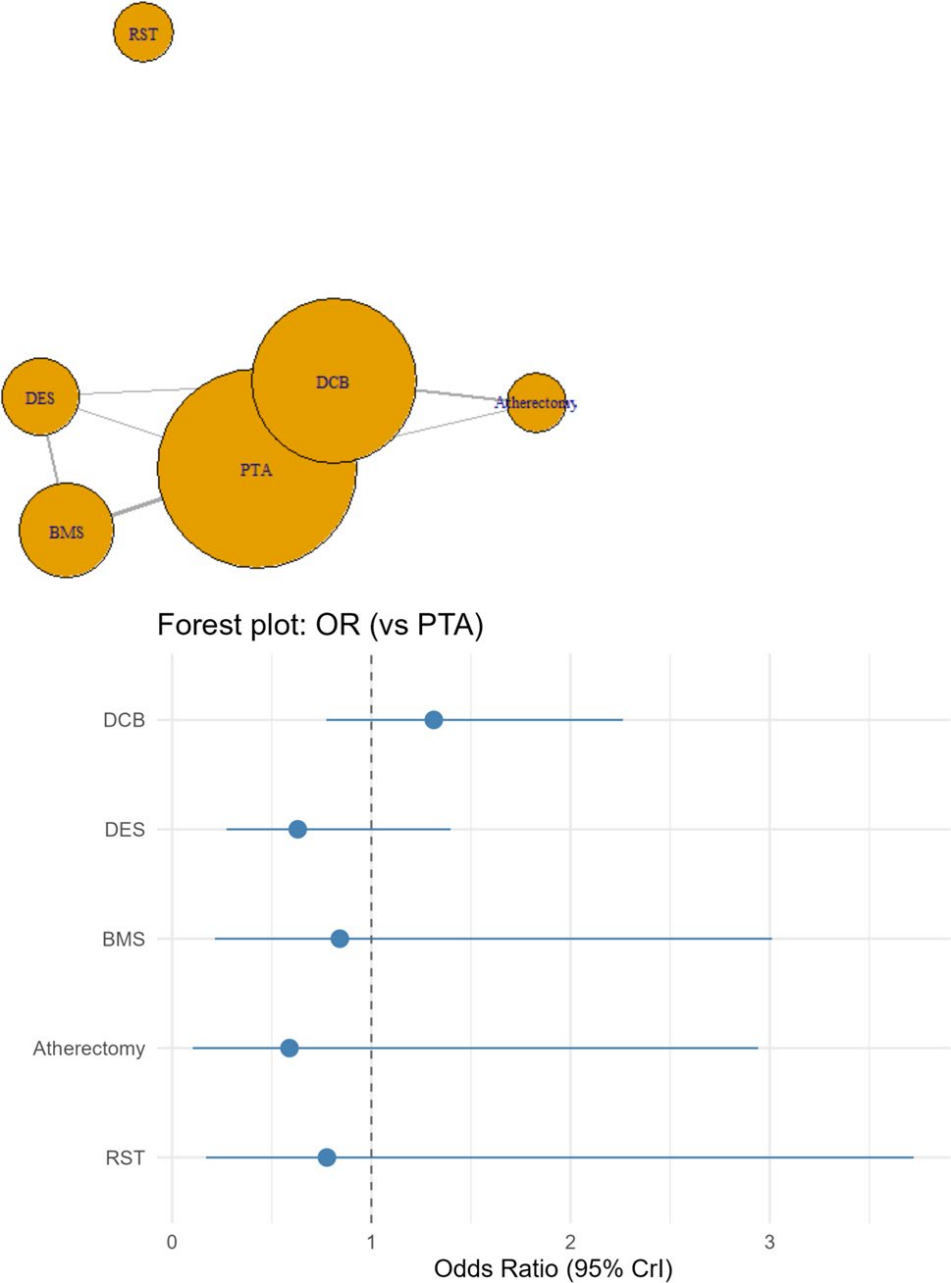
Network plot for the secondary outcome of Major amputation at 6 months, which depicts a well-connected network except for RST, which only had single-arm studies, and a forest plot depicting odds ratios (ORs) of major amputation at 6 months for the interventions of interest compared with PTA

Compared with PTA (Fig. 5), DES (OR: 0.63, 95% CrI 0.27, 1.39), BMS (OR: 0.84, 95% CrI 0.21, 3.01), Atherectomy (OR: 0.59, 95% CrI 0.1, 2.94), and RST (OR: 0.77, 95% CrI 0.17, 3.72) were all associated with statistically insignificant lower odds of major amputation at 6 months. DCB was associated with statistically insignificant higher odds of a major amputation at 6 months (OR: 1.31, 95% CrI 0.77, 2.26).

According to SUCRA values (Supplementary Table 1D), DES had the highest probability of being the best treatment (70%), followed by Atherectomy (66%), RST (54%), BMS (50.7%), PTA (40%), and DCB (18.2%).

### Meta-Regression

In the meta‑regression analyses (Table 2), CKD was associated with a 56% increase in odds of CD-TLR at 6 months, while longer lesion length conferred a similar 56% increase, whereas no covariates, including age, diabetes, smoking status, CKD, and lesion length, predicted 30‑day MAE, 12‑month mortality, or risk of major limb amputations at 6 months.

### Publication Bias

Funnel Plots (Supplementary Figs. 1 A–D) were generated to visually assess for publication bias that revealed asymmetry with relatively larger negative effects produced by smaller studies, confirmed with Egger’s test (Supplementary Fig. 1 A–D), which was consistent across all the outcomes.

### Sensitivity Analysis

To evaluate the robustness of the primary findings and account for potential publication bias, sensitivity analyses were performed across all outcomes. These analyses excluded studies identified as contributing disproportionately to funnel plot asymmetry based on Egger’s regression results and visual inspection.

### Primary Outcome

For the primary outcome of MAE at 30 days, DEEPERREVEAL was excluded. Sensitivity analysis (Supplementary Fig. 2) yielded identical odds ratios and credible intervals to the primary analysis, indicating that the results are robust to variations in model assumptions and study inclusion criteria.

### Secondary Outcomes

Sensitivity analyses across all the secondary outcomes (Death at 12 months, CD-TLR at 6 months, and major amputations at 6 months) yielded similar results compared to the primary analyses (Supplementary Fig. 3A–C). Specifically, studies contributing the most towards plot asymmetry (DEEPER, OPTIMIZEBTK, and PADI) were excluded based on their combination of high standard errors and extreme treatment effects, which had suggested small-study effects. After exclusion, the odds ratios and confidence intervals remained consistent with the primary analysis across all endpoints, indicating that the overall findings were stable and not significantly influenced by these studies.

### Risk of Bias Assessment

**Fig. 6:**
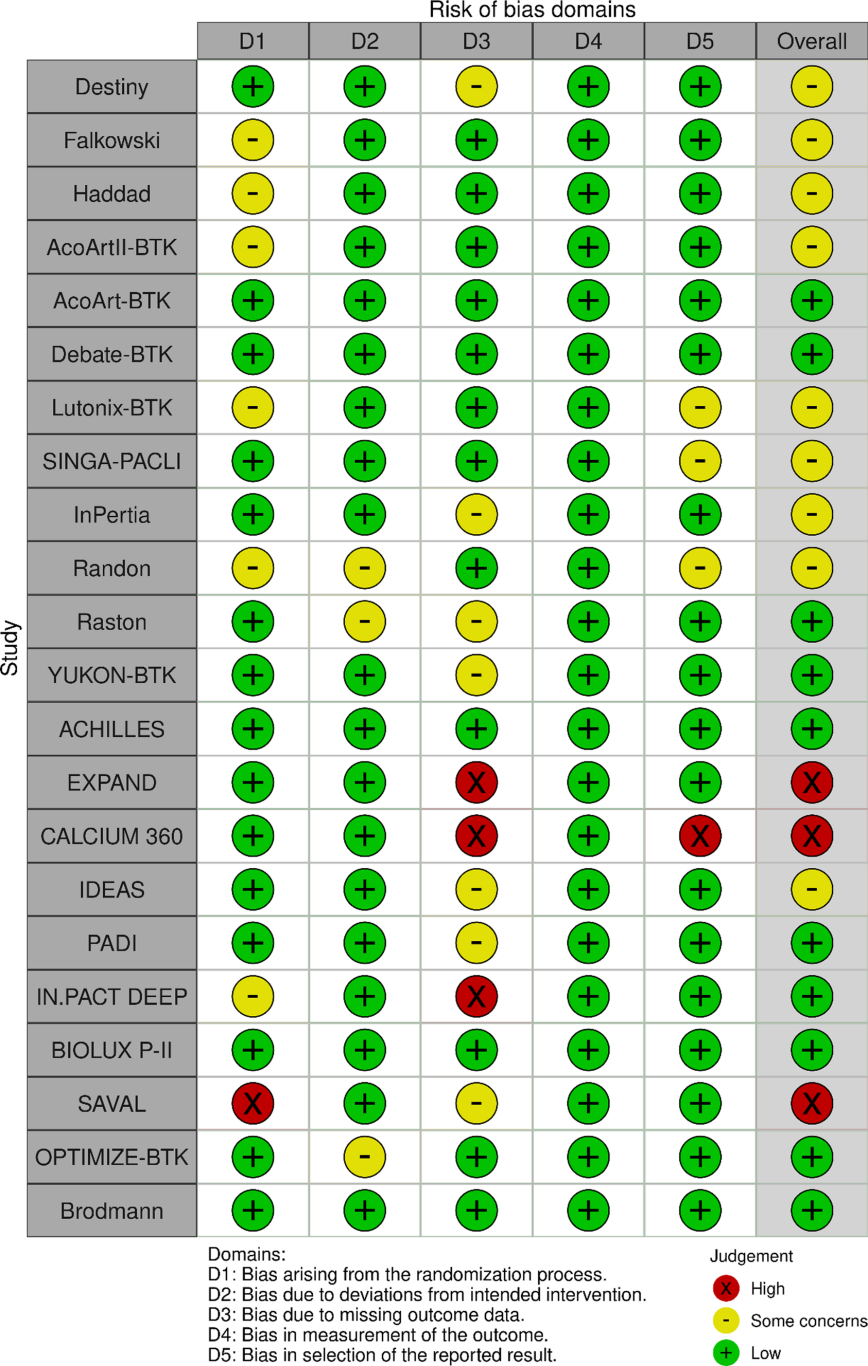
Risk of Bias Assessment for randomized studies: according to the Cochrane tool RoB 2 (Traffic light plot). [48]

**Fig. 7:**
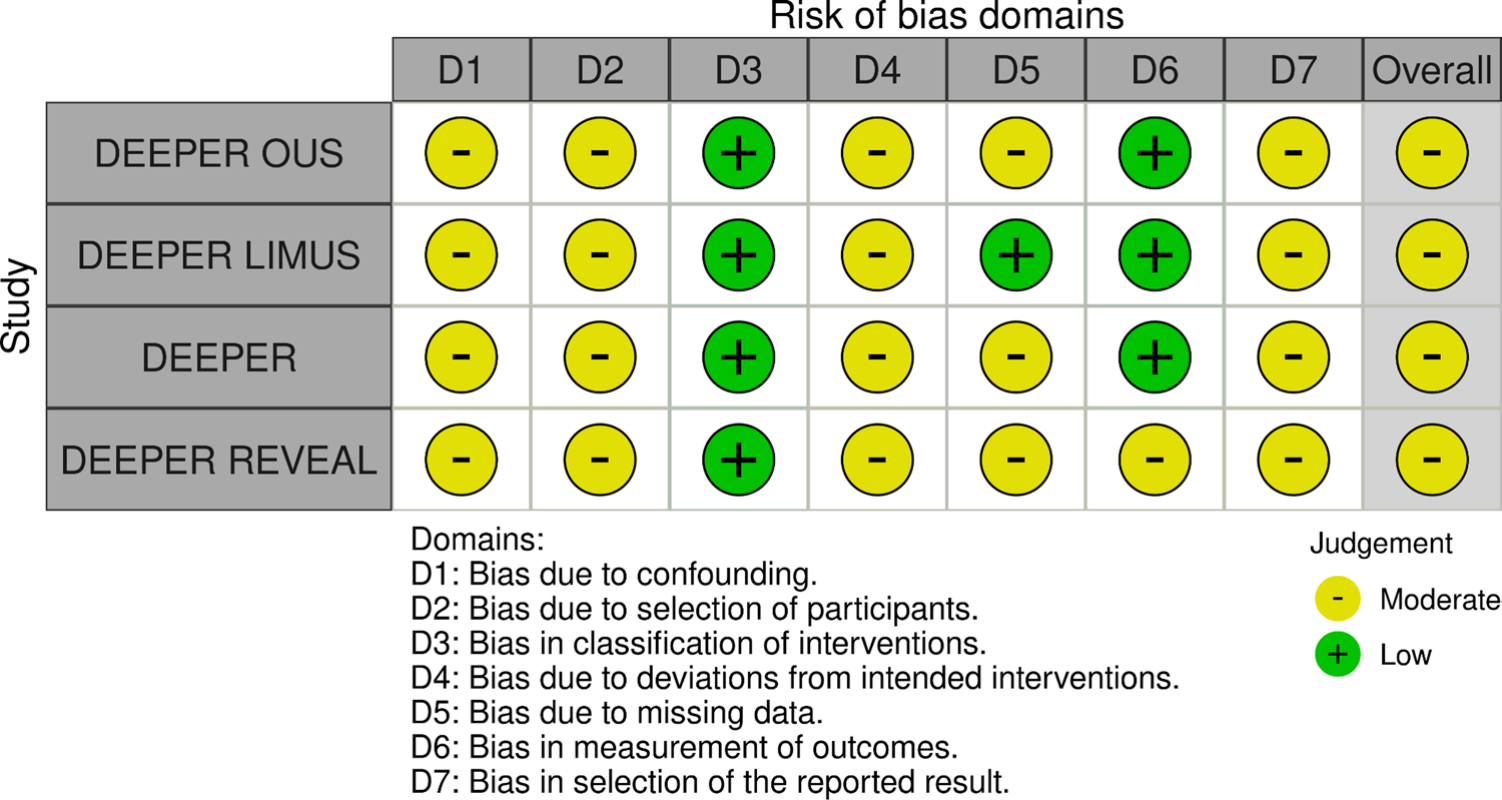
Risk of Bias Assessment for non-randomized studies: according to the Cochrane tool ROBINS-I (Traffic light plot)

Risk of bias was assessed using the Cochrane Risk of Bias 2 (ROB 2) tool for randomized controlled trials (Fig. 6) and the ROBINS-I tool for non-randomized studies (Fig. 7). Overall, most studies were judged to be at low risk of bias or to have some concerns (DESTINY, Falkowski, Haddad, AcoArtII-BTK, Lutonix-BTK, SINGA-PACLI, InPertia, Randon, IDEAS), with only a small number demonstrating high risk of bias (EXPAND, CALCIUM 360, SAVAL), primarily due to the open-label nature of interventions and incomplete angiographic follow-up, despite adequate randomization procedures and objective outcome assessment by independent core laboratories. All non-randomized studies were judged to be at moderate risk of bias, driven predominantly by unavoidable confounding inherent to single-arm, non-randomized designs without concurrent control groups. Across study designs, outcome measurement was generally robust, with prespecified endpoints and independent adjudication. Overall, the risk of bias across included studies was considered acceptable for pooled analysis.

## Discussion

This study is the largest network meta-analysis of infra-popliteal artery interventions, including 21 RCTs and 4 single-arm studies with a total of 3184 patients. Compared to existing literature, this is the first network meta-analysis study that includes single-arm studies on Retrievable Scaffold Therapy. Our network meta-analysis showed no statistically significant differences in 30-day major adverse events (MAEs) among treatment modalities when compared with plain balloon angioplasty (PTA). Point estimates varied across interventions, but the associated uncertainty intervals overlapped the null, indicating no evidence of differential short-term safety. Although SUCRA rankings suggested higher probabilities of favorable ranking for balloon angioplasty followed by atherectomy and DES, these rankings should be interpreted as relative probabilities rather than evidence of superiority, given the lack of statistically significant comparisons.

Infrapopliteal artery disease exhibits distinct histopathological features that complicate endovascular therapy. Compared with coronary disease, infrapopliteal lesions are typically long, diffuse, and extensively calcified, frequently presenting as chronic total occlusions [49]. Calcific involvement of both the intimal and medial layers is common, and advanced disease may demonstrate osteogenic remodeling of the arterial wall. These features are particularly prominent in chronic limb-threatening ischemia, where diabetes mellitus and chronic kidney disease further intensify the extent and severity of vascular calcification [49, 50]. In contemporary practice, PTA remains the most frequently used endovascular approach for BTK revascularization. However, vessel injury, elastic recoil, and flow-limiting dissections contribute to neointimal proliferation and high restenosis rates, limiting the durability of balloon-only strategies [51, 52]. Although BMS can address acute recoil and dissections, restenosis remains common, restricting their use primarily to bail-out settings. Consistent with these limitations, our meta-analysis demonstrates no clear advantage of BMS over PTA alone. In contrast, drug-based technologies, including DES and DCB, aim to inhibit neointimal hyperplasia through localized antiproliferative drug delivery [53, 54].

Previous NMAs have shown that evidence supporting DES in BTK disease is largely derived from balloon-expandable coronary platforms, which demonstrated improved short- and mid-term primary patency compared with PTA or BMS, without consistent reductions in major amputation or mortality. These benefits attenuate with increasing lesion length and appear device-specific, as demonstrated in SAVAL [34]. Although our NMA includes the same DES trials as prior analyses, it extends existing evidence by emphasizing clinically relevant outcomes. DES exhibited a significant reduction in clinically driven target lesion revascularization at 6 months, consistent with reduced restenosis, yet without a statistically significant reduction in major amputation. Collectively, these findings position DES as a strategy that may reduce early reintervention without durable benefits in survival or limb salvage, reinforcing the need for device- and lesion-specific evaluation in BTK interventions.

DCB technology offers theoretical advantages for BTK revascularization, including the absence of a permanent metallic scaffold and suitability for long lesions and anatomic flexion points where stent durability is limited [55, 56]. Randomized evidence in BTK disease, limited to paclitaxel-coated DCBs, has been heterogeneous. Early safety concerns arose from the IN.PACT DEEP trial, which failed to demonstrate superiority over PTA and reported higher amputation rates, leading to withdrawal of the IN.PACT Amphirion device despite neutral long-term outcomes [29]. Subsequent trials yielded mixed efficacy signals, with BIOLUX P-II [27] showing numerically improved patency, the Lutonix BTK trial [28] demonstrating short-term benefit not sustained beyond 12 months, and DEBATE-BTK [25] and ACOART trials [26] reporting more favorable reintervention outcomes. In oir NMA, DCB ranked lowest for safety outcomes by SUCRA, with no mortality or major amputation benefit at follow-up. Although DCB demonstrated a significant reduction in CD-TLR at 6 months, this did not translate into improved limb salvage.

Adjunctive atherectomy has been proposed as a strategy to modify calcified BTK lesions by reducing plaque burden, improving vessel compliance, and potentially enhancing the effectiveness of subsequent balloon-based therapies and drug uptake [50]. Despite these theoretical advantages, the randomized evidence base for atherectomy in BTK interventions remains limited. Prior NMAs suggested that atherectomy used in combination with PTA or DCB frequently ranked favorably for outcomes such as primary patency and target lesion revascularization; however, these conclusions were largely driven by SUCRA rankings rather than statistically robust treatment effects [57]. In our analysis, Atherectomy-based approaches demonstrated the highest probability of ranking among the most effective strategies for reducing repeat revascularization. In addition, it also showed a statistically significant association with reduced 12-month mortality relative to PTA and a lower risk of CD-TLR at 6 months.

Compared to DES, DCB, or PTA, retrievable scaffold therapy (RST) offers the advantage of providing temporary mechanical support without leaving a permanent implant, addressing issues such as neointimal hyperplasia, stent fracture, and limited drug delivery in heavily calcified or complex BTK lesions. While DES and DCB improve patency through drug elution, their effectiveness can be hindered by rigid calcified segments, and PTA alone offers no long-term structural support. Preclinical studies in porcine models demonstrated that temporary spiked scaffolds facilitate drug delivery into calcified vessel walls while minimizing neointimal proliferation and lumen loss, in contrast to permanent stents, which induced significant neointimal thickening [58]. Clinical evidence for RST remains limited but promising. In one trial from the University Herzzentrum Freiburg, 12-month target lesion primary patency was 74.4% overall and 75% at the single center, while CD-TLR was 89.5% overall and 83% at the single center. Rutherford class improved across 12 months, and approximately 60% of wounds in the overall cohort and 50% at the single center achieved complete healing. Freedom from major amputation at 12 months was 98.9% overall and 100% at the single center [47]. In a second multicenter study, RST deployment was successful in all cases with 100% device and procedural success, negligible acute vessel recoil, and no in-hospital major adverse events. Primary patency at 6 months was 95.2% by angiography and 91.3% by duplex ultrasound, remaining 89.5% at 12 months. Rutherford class improved in 68% of patients, and WIfI wound scores decreased, with no patient at high risk for amputation at 12 months [45]. Our NMA is the first to synthesize this data and evaluate RST against the major endovascular strategies described above for BTK arterial disease. RST was associated with numerically lower 30-day major adverse events (OR 0.73) and lower odds of CD-TLR at 6 months (OR 0.42) compared with PTA, although not statistically significant. SUCRA rankings indicated moderate probability of being the best intervention for reducing major adverse events (53.6%) and preventing reintervention (64%), with comparable major amputation rates at 6 months (OR 0.77; SUCRA 54%). These findings suggest RST may address key limitations of existing BTK devices, especially in calcified and challenging lesions, but randomized studies are needed to confirm its comparative efficacy and safety.

Meta-regression analyses identified chronic kidney disease and longer lesion length as variables associated with higher odds of CD-TLR at 6 months, each conferring a 56% increase. No associations were observed between evaluated covariates and 30-day MAEs, 12-month mortality, or major amputation at 6 months. As recommended by PRISMA-NMA, these analyses are exploratory and intended to assess potential sources of heterogeneity rather than to inform individualized treatment selection.

This analysis is subject to an important limitation related to the differential availability of evidence across treatment strategies. Specifically, retrievable scaffold therapy (RST) was informed exclusively by single-arm observational studies, whereas other interventions were supported by randomized or comparative trial data. This approach reflects the current state of the literature rather than selective inclusion, as no randomized or comparative studies of RST meeting the prespecified eligibility criteria were available. Excluding these studies would have resulted in the structural omission of a prespecified intervention class and precluded any quantitative assessment of its outcomes. Accordingly, prospective observational RST studies were retained and incorporated using a Bayesian arm-based framework, in which single-arm data contribute to the estimation of absolute event risks rather than direct comparative effects. In contrast, observational studies were not included for other interventions because randomized evidence was available, and restricting those treatment nodes to trial data reduced heterogeneity and preserved internal validity. Nonetheless, the absence of randomized evidence for RST limits assessment of transitivity and consistency and introduces potential for residual confounding and selection bias that cannot be fully addressed in aggregate-level analyses. As such, comparative findings involving RST should be interpreted with particular caution and viewed as exploratory, underscoring the need for rigorously conducted randomized or comparative studies to better define its role relative to established endovascular therapies.

## Conclusion

Ongoing advances in infrapopliteal endovascular therapy continue to target the biological and mechanical limitations that have constrained durable outcomes in BTK disease. While recent innovations in drug-coated balloon and drug-eluting stent technology aim to optimize antiproliferative efficacy, therapeutic success remains fundamentally limited by inefficient drug transfer and subtherapeutic tissue retention, particularly in calcified and complex lesions. Retrievable scaffold therapy represents a conceptual evolution in this paradigm, providing temporary luminal support and controlled vessel expansion that may improve drug uptake and mitigate recoil without promoting chronic inflammatory responses, stent fracture, or neointimal hyperplasia, and has so far shown promising clinical outcomes. Future randomized studies incorporating lesion-specific stratification, optimized drug–device combinations, and clinically meaningful endpoints will be critical to define the role of such hybrid approaches and to inform individualized treatment algorithms for BTK revascularization in CLTI.

## AI Disclosure

During the preparation of this work, the author(s) used Grammarly.ai in order to improve language and readability. After using this tool/service, the author(s) reviewed and edited the content as needed and take full responsibility for the content of the published article.

## Supplementary Information

Below is the link to the electronic supplementary material.

Supplementary file1 (DOCX 4347 kb)
