# Supplementary material for: Endovascular Treatment Modalities for Infrapopliteal Artery Disease: A Bayesian Network Meta-Analysis with Exploratory Evaluation of Retrievable Scaffold Therapy
Source: Cardiovasc Intervent Radiol. 2026 Apr 30;49(6):1079–93. doi: 10.1007/s00270-026-04452-0 (PMC13212623; doi:10.1007/s00270-026-04452-0)
Supplement: Supplementary file 1 — Supplementary file1 (DOCX 4347 kb) [file 270_2026_4452_MOESM1_ESM.docx]

**Supplementary Table 1 A-D: SUCRA values for MAE (A), Death (B), CD-TLR (C ), and Major Amputations (D).**

**A.**

| Treatment | SUCRA |
| --- | --- |
| Atherectomy | 0.7722066667 |
| BMS | 0.63122 |
| DES | 0.6138866667 |
| RST | 0.53616 |
| PTA | 0.3552933333 |
| DCB | 0.09123333333 |

**B.**

| Treatment | SUCRA |
| --- | --- |
| Atherectomy | 0.9786066667 |
| PTA | 0.5529733333 |
| RST | 0.5268466667 |
| DCB | 0.4386533333 |
| BMS | 0.37748 |
| DES | 0.12544 |

**C.**

| Treatment | SUCRA |
| --- | --- |
| Atherectomy | 0.8847333333 |
| RST | 0.6414933333 |
| DCB | 0.63274 |
| DES | 0.6279066667 |
| PTA | 0.2003133333 |
| BMS | 0.01281333333 |

**D.**

| Treatment | SUCRA |
| --- | --- |
| DES | 0.7062333333 |
| Atherectomy | 0.6648 |
| RST | 0.5388066667 |
| BMS | 0.5079866667 |
| PTA | 0.4003666667 |
| DCB | 0.1818066667 |

**Supplementary Table 2: Meta-Regression Analysis**

| Column 1 | Column 2 | Column 3 | Column 4 |
| --- | --- | --- | --- |
| **MAE at 30 days** |  |  |  |
| Covariate | Beta_Median | CI_Lower | CI_Upper |
| Mean age | -0.4580783951 | -1.069108036 | 0.1917947636 |
| Diabetes | -0.08206799155 | -1.019982803 | 1.024474595 |
| smoker | -0.590301692 | -1.777167569 | 0.5722192511 |
| CKD | 0.0517370328 | -1.202607062 | 0.9658497954 |
| Lesion length | -0.6953083678 | -1.709732331 | 0.2354573431 |
|  |  |  |  |
| **Death at 12 months** |  |  |  |
| Covariate | Beta_Median | CI_Lower | CI_Upper |
| Mean age | -0.1012717245 | -0.3178685441 | 0.143603139 |
| Diabetes | -0.1380049718 | -0.4556246473 | 0.1344098438 |
| smoker | -0.005880943597 | -0.3194320339 | 0.3621577618 |
| CKD | 0.07754685597 | -0.2077614705 | 0.3655025386 |
| Lesion length | -0.1604642058 | -0.4684330379 | 0.161624559 |
|  |  |  |  |
| **CD-TLR at 6 months** |  |  |  |
| Covariate | Beta_Median | CI_Lower | CI_Upper |
| Mean age | -0.2963706529 | -0.6117594345 | 0.01060946223 |
| Diabetes | -0.2863741313 | -0.7605051418 | 0.1043872151 |
| smoker | 0.02318662612 | -0.5001876996 | 0.4768791248 |
| CKD | 0.4450807827 | 0.04627050554 | 0.9021873198 |
| Lesion length | 0.4484038995 | 0.03881403179 | 0.9361920302 |
|  |  |  |  |
| **Major Amputation at 6 months** |  |  |  |
| Covariate | Beta_Median | CI_Lower | CI_Upper |
| Mean age | -0.2277165123 | -0.7975579082 | 0.4499664126 |
| Diabetes | 0.4809028507 | -0.4780611729 | 1.480985342 |
| smoker | -0.7668943294 | -1.947475899 | 0.2947943268 |
| CKD | -0.1457120142 | -1.096617563 | 0.8525202272 |
| Lesion length | -0.5683678262 | -1.805707201 | 0.5052088846 |

Table 2: Meta‑regression analyses across outcomes. No covariates were significantly associated with 30‑day MAE, 12‑month mortality, or 6‑month major amputation. In contrast, CKD (β = 0.45, 95% CI 0.05–0.90) and lesion length (β = 0.45, 95% CI 0.04–0.94) were independently associated with a higher risk of CD‑TLR at 6 months, while other patient characteristics did not consistently modify outcomes

**Supplementary Figure 1. Funnel Plot and Egger’s plot to Detect Publication bias for MAE (A), Death (B), CD-TLR at 6 months (C ), and Major Amputations (D).**

**A.**

**
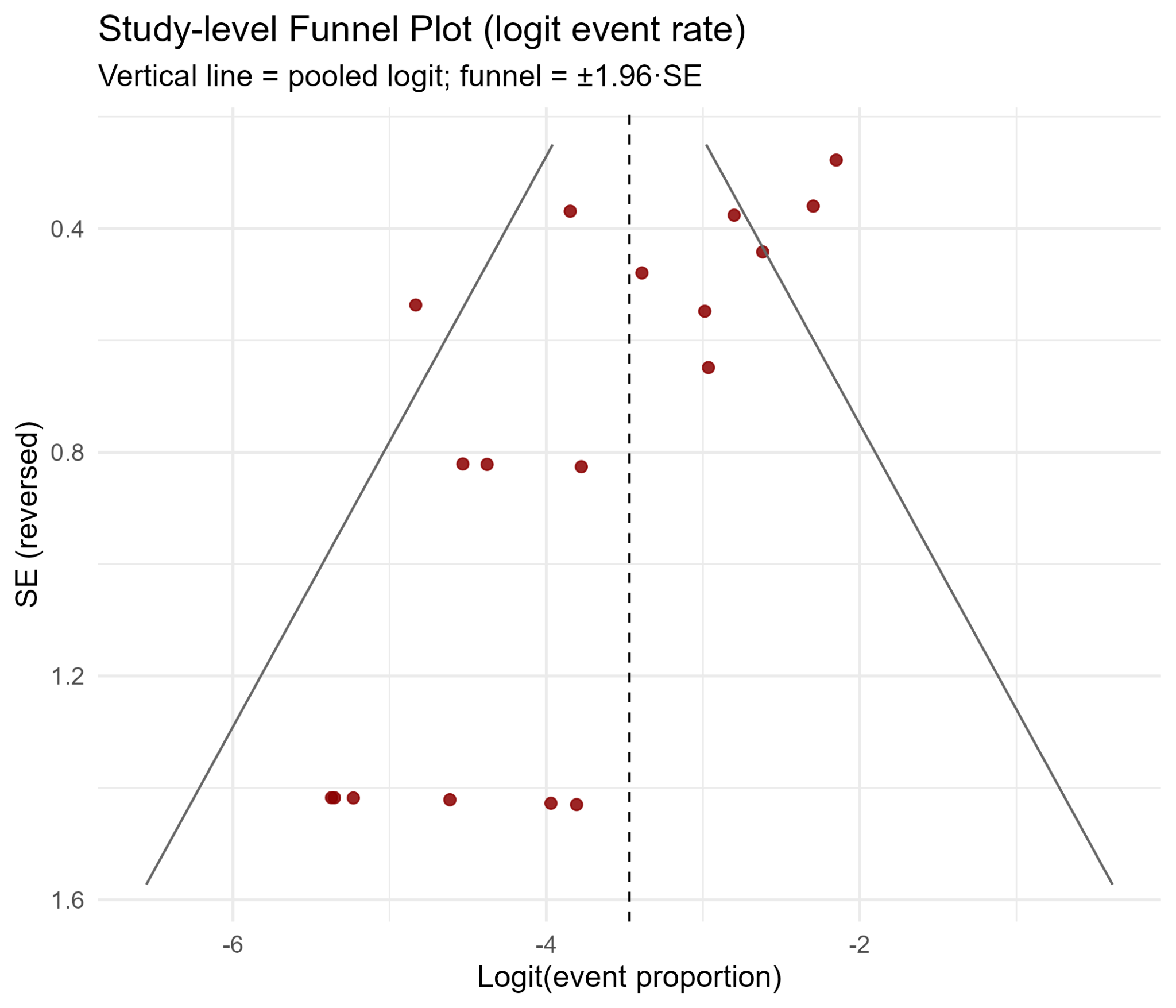

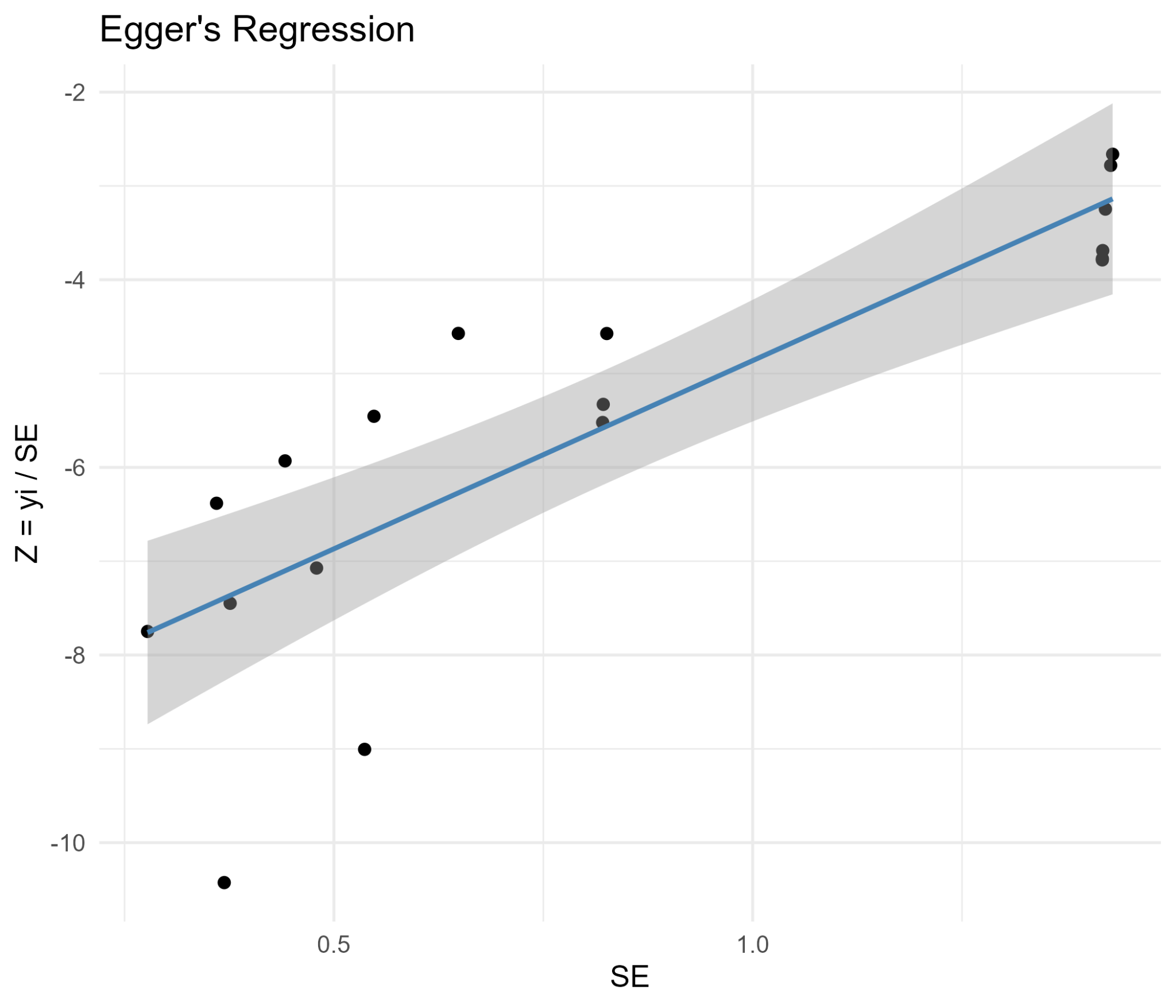
**

**B.**

**
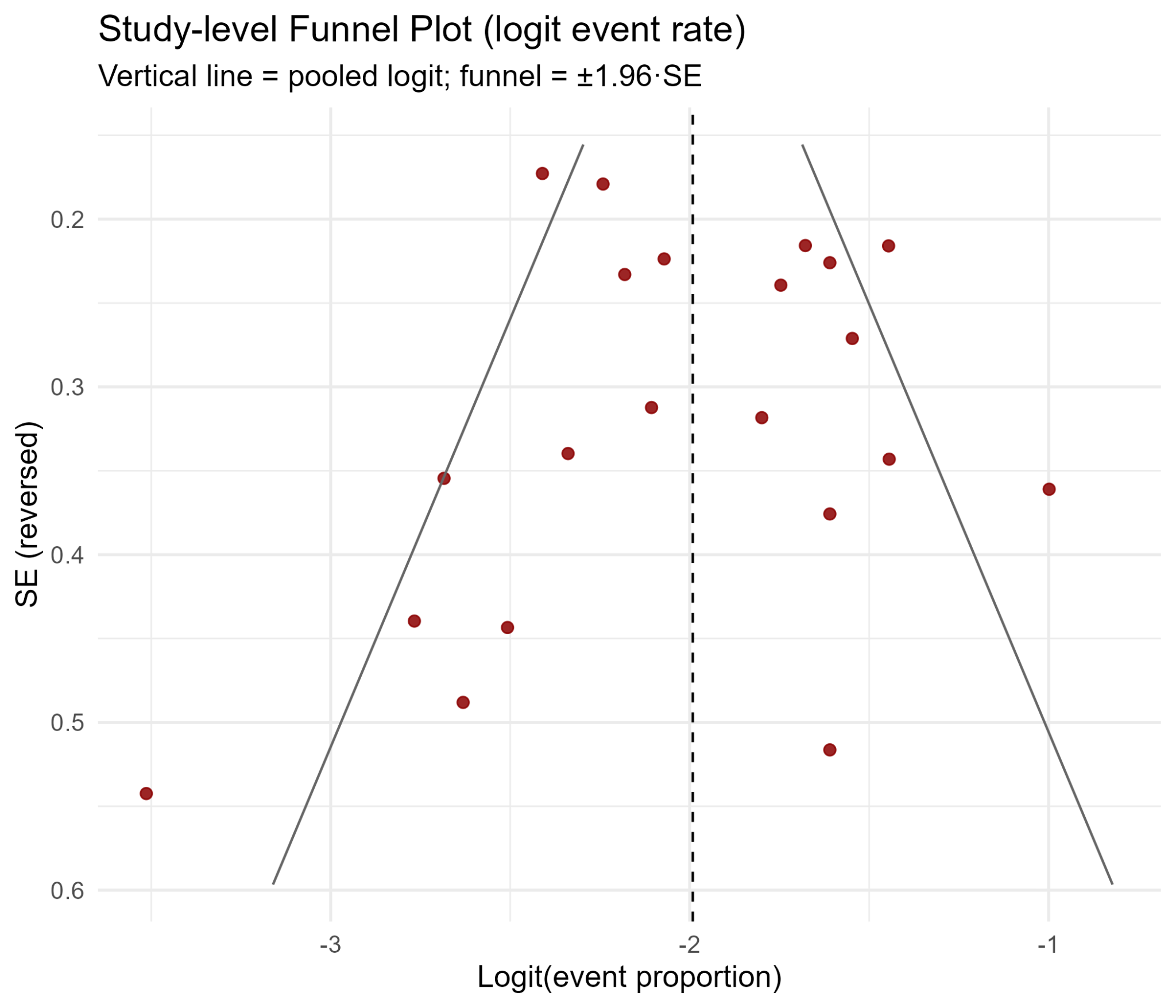
**

**
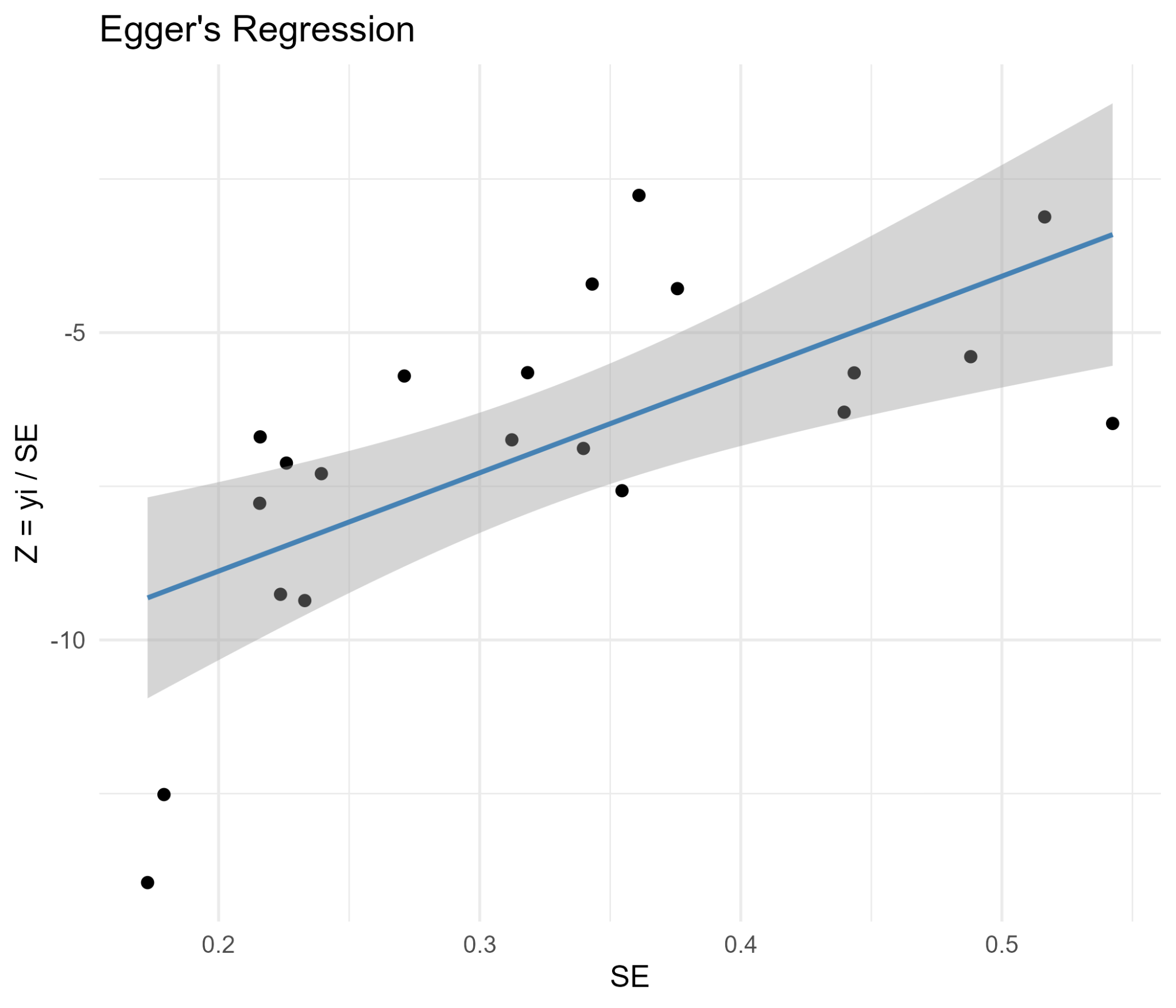
**

**C.**

**
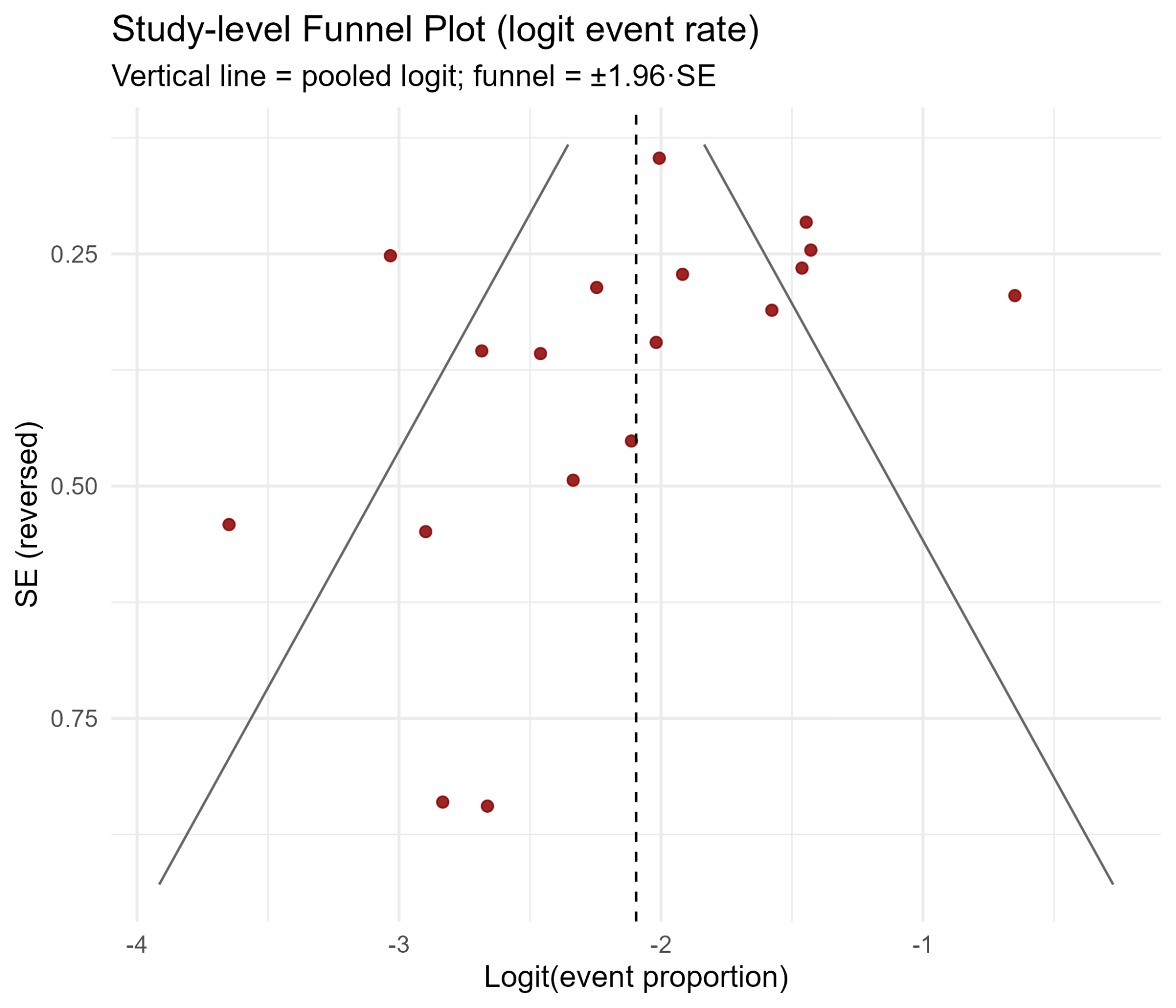
**

**
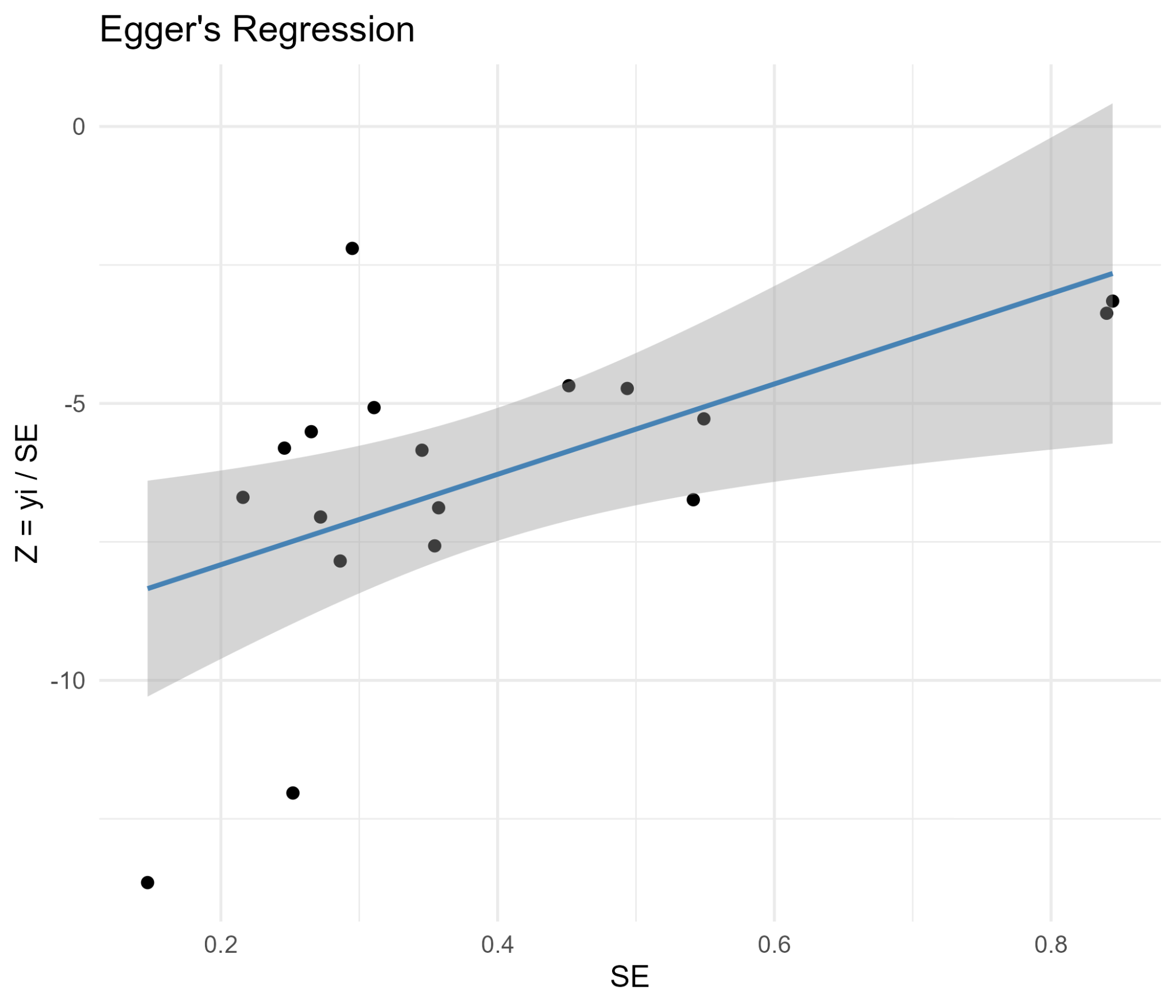
**

**D.**

**.
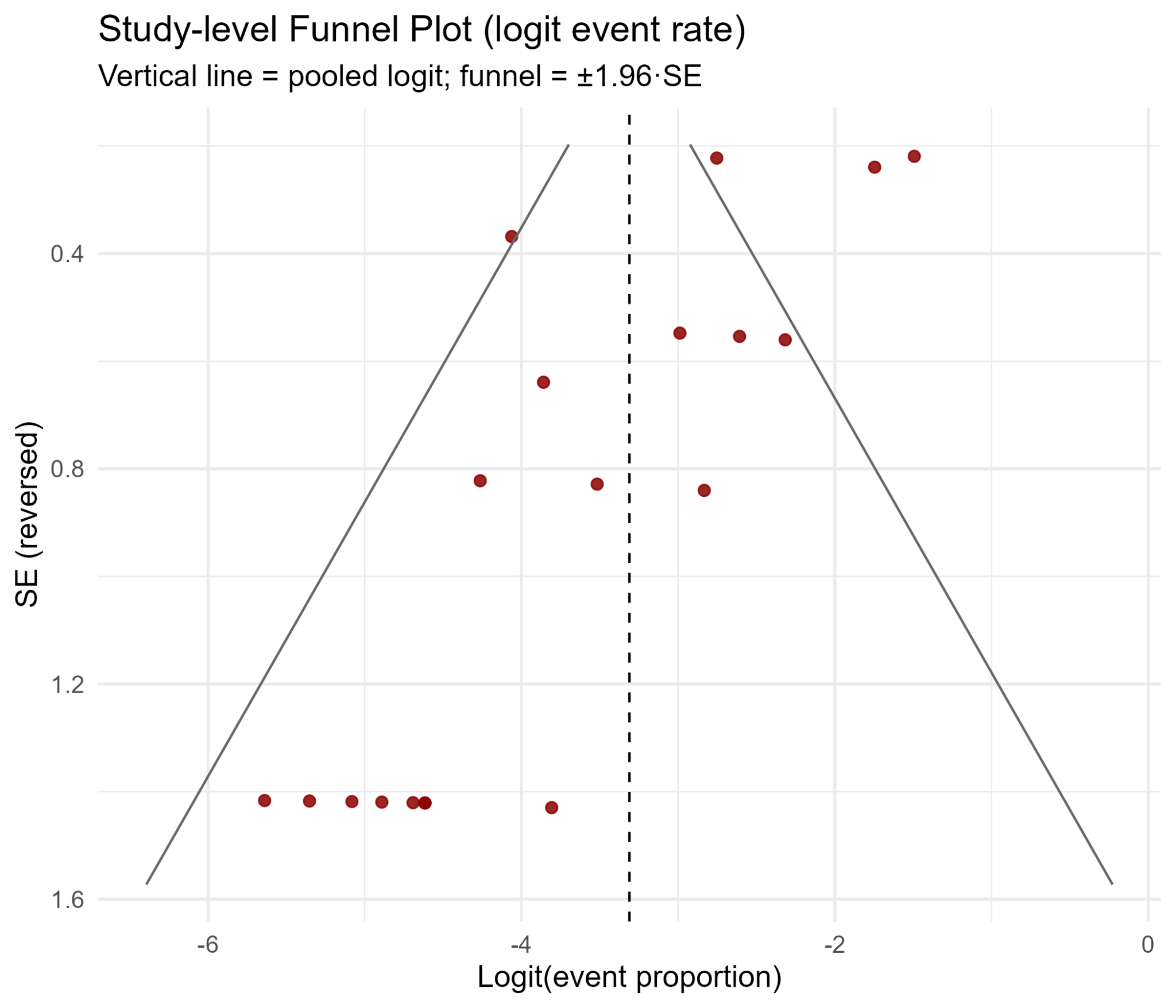
**

**
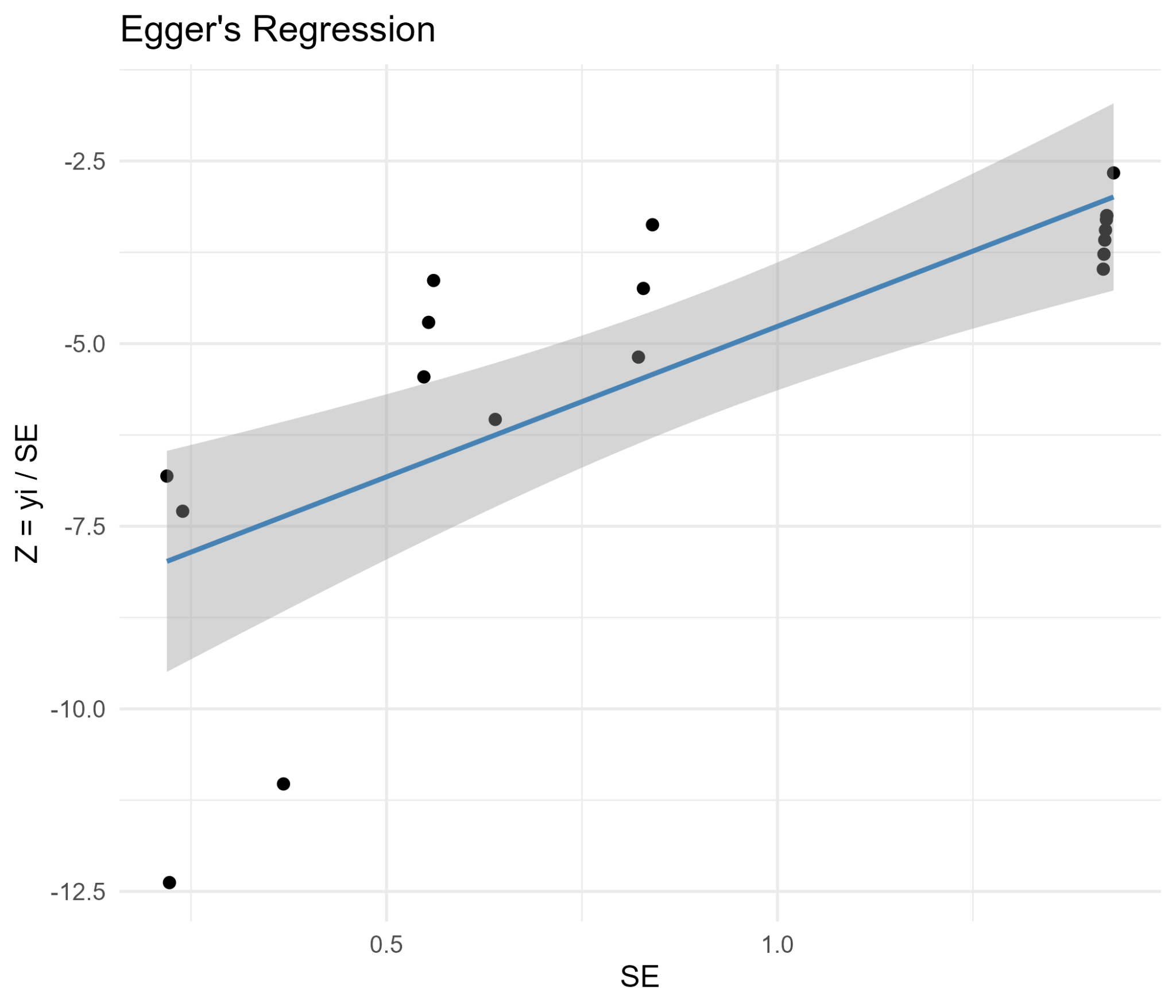
**

**Supplementary Figure 2: Forest plot for the sensitivity analysis of MAE at 30 days


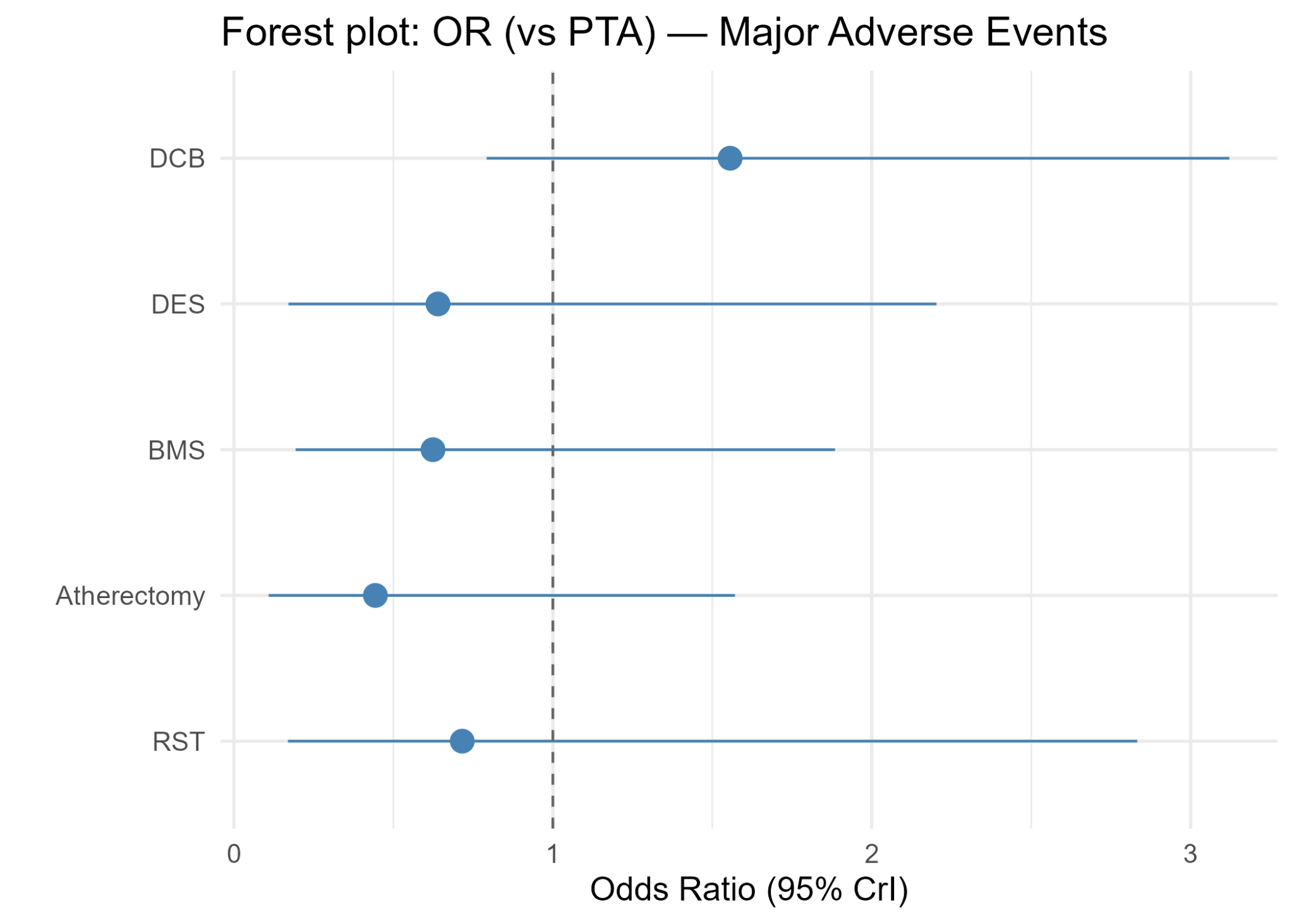
**

**Supplementary Figure 3: Sensitivity Analysis for the secondary outcomes of Death (A), CD-TLR at 6 months (B), and major amputations (C).**

**A.**

**
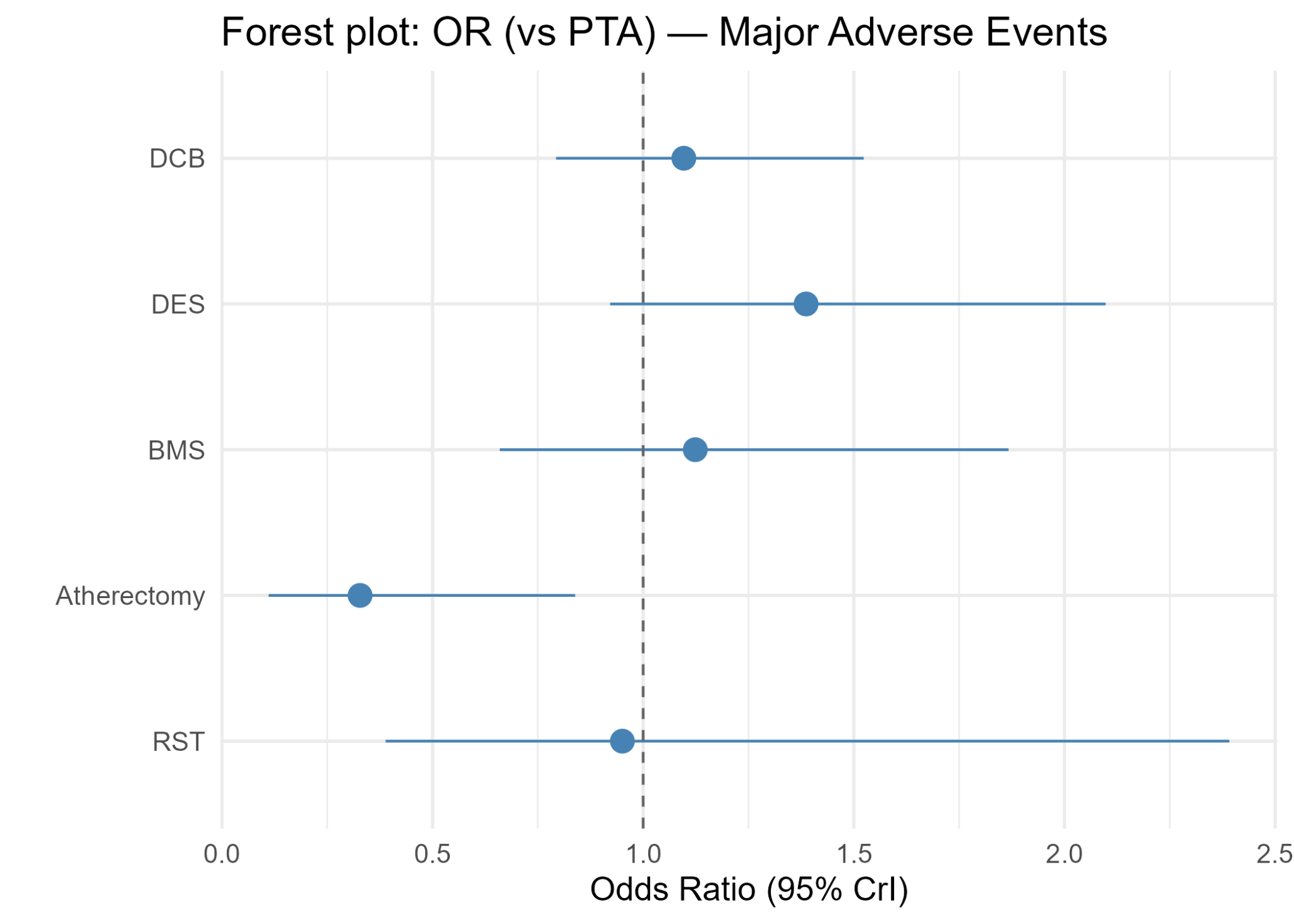
**

**B.**

**
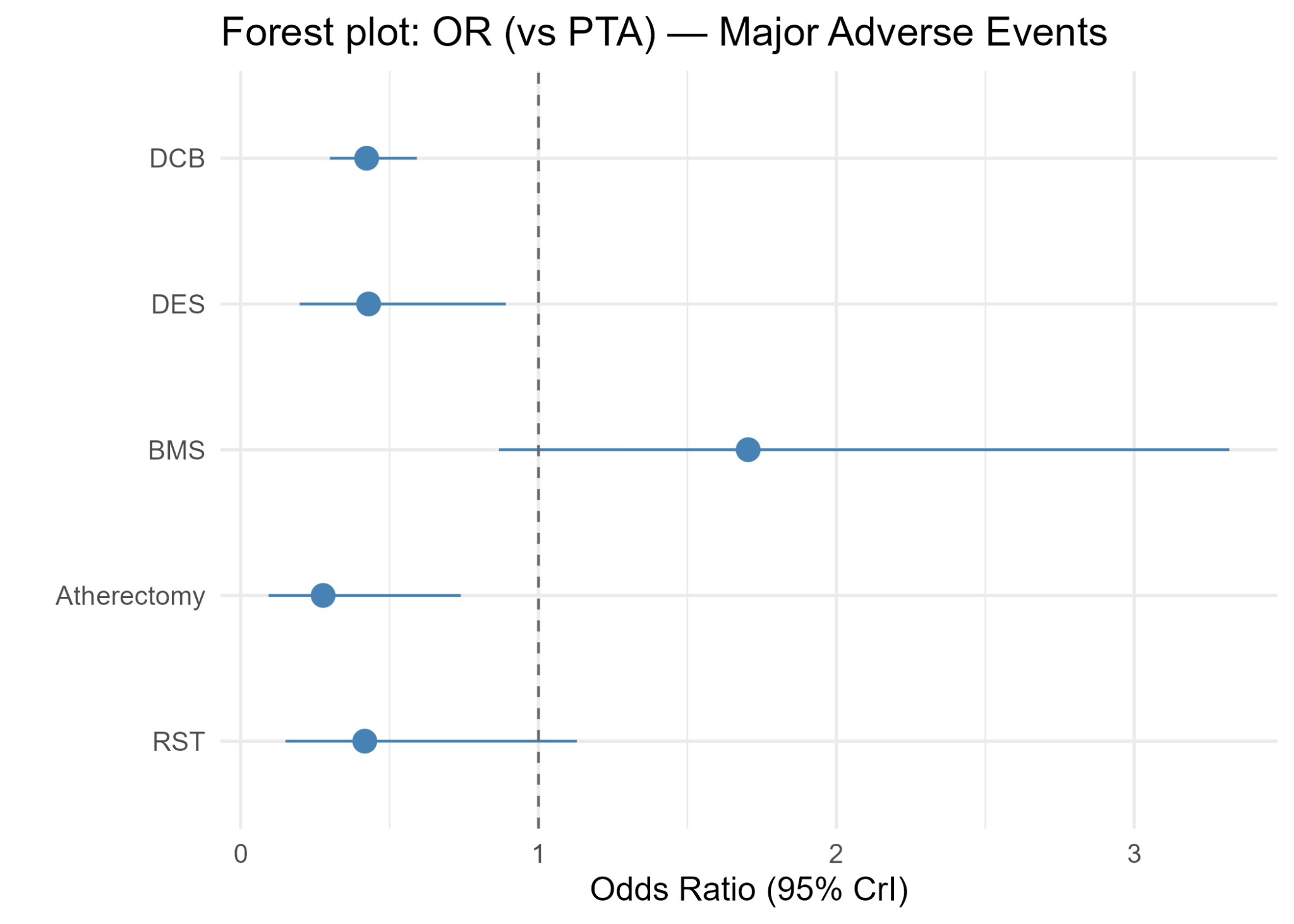
**

**C.**

**
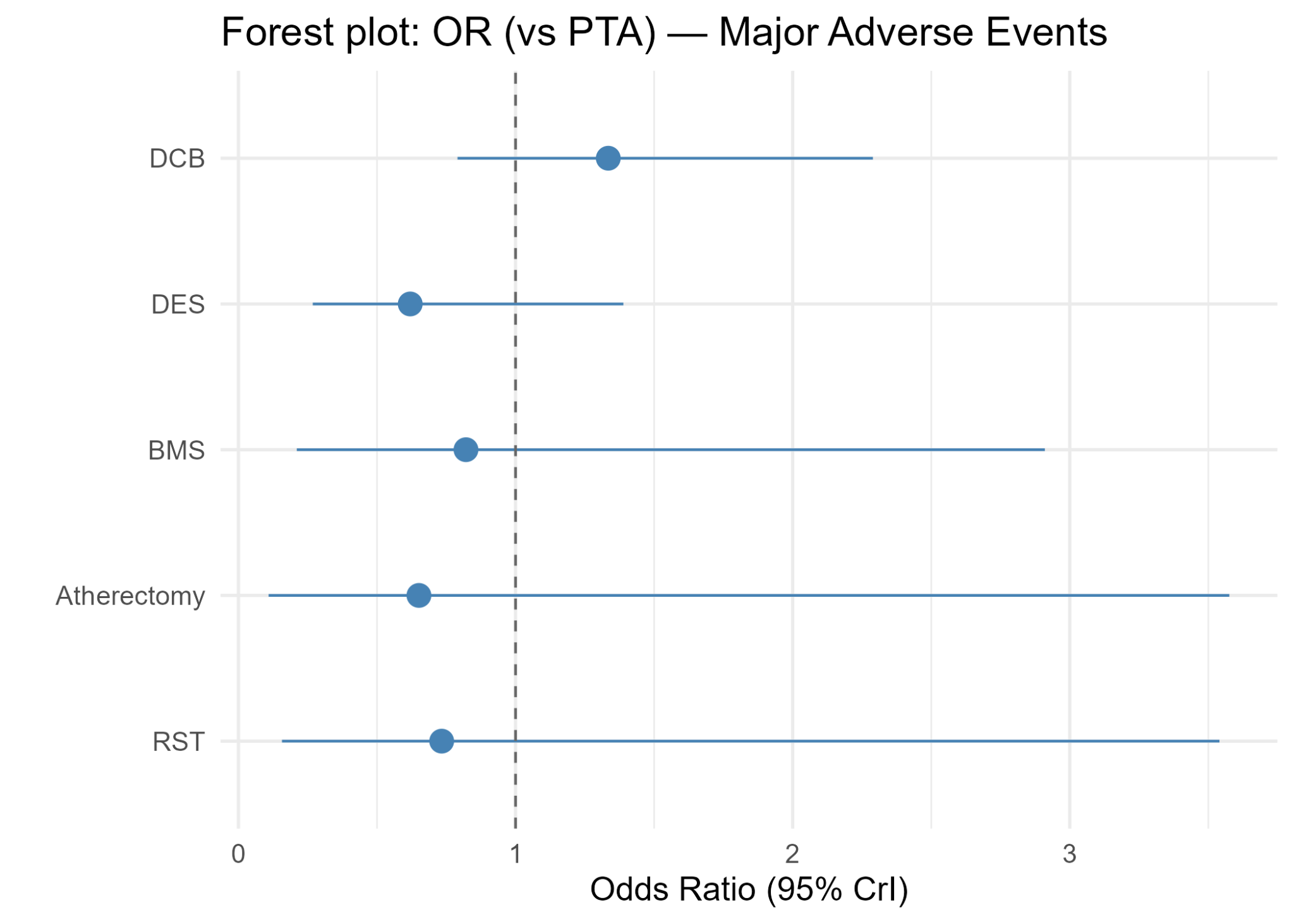
**

**Supplementary Figure 4:**


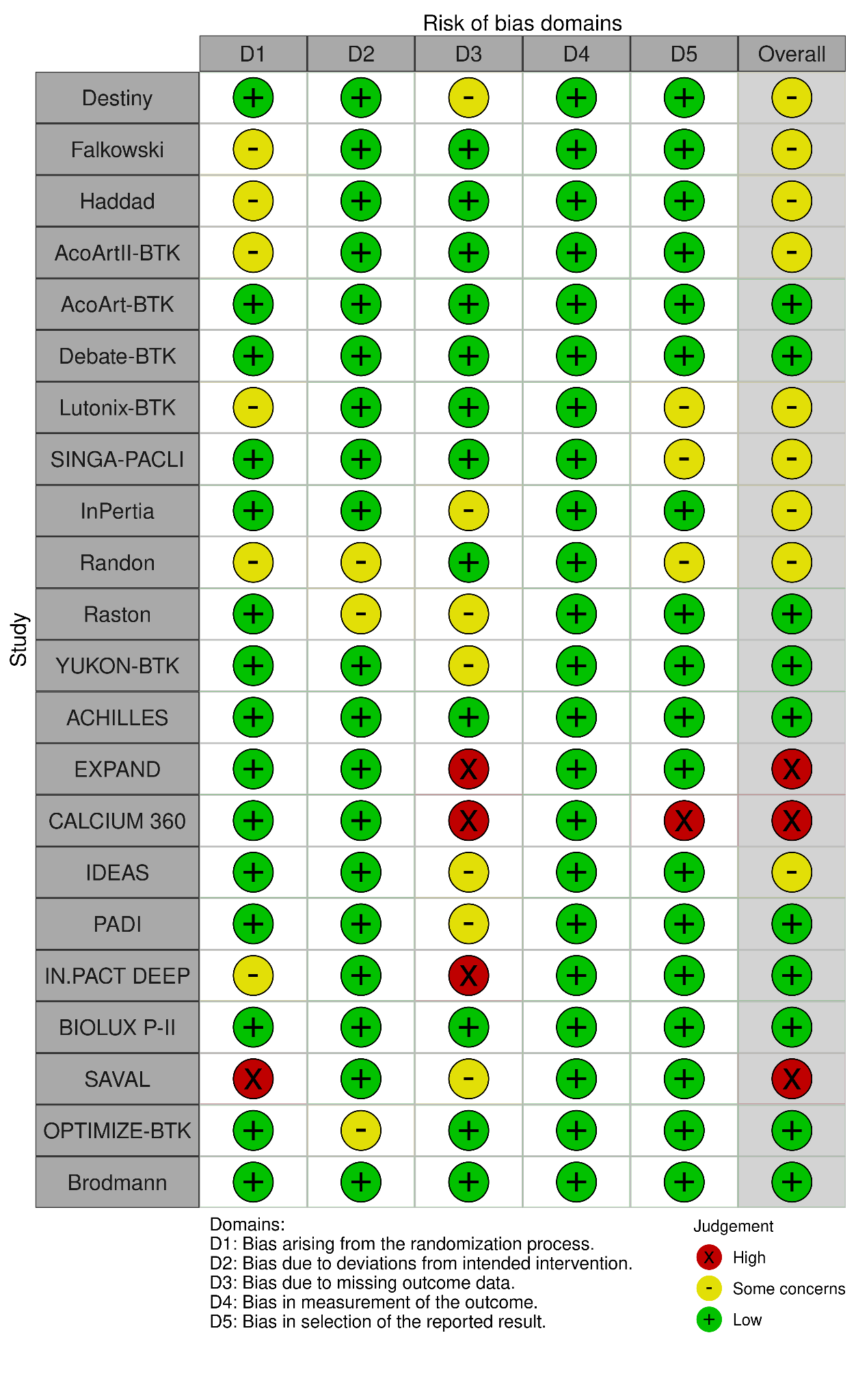


Supplementary Fig 4: Risk of Bias Assessment for randomized studies: according to the Cochrane tool RoB 2 (Traffic light plot)^48^

**Supplementary Figure 5**


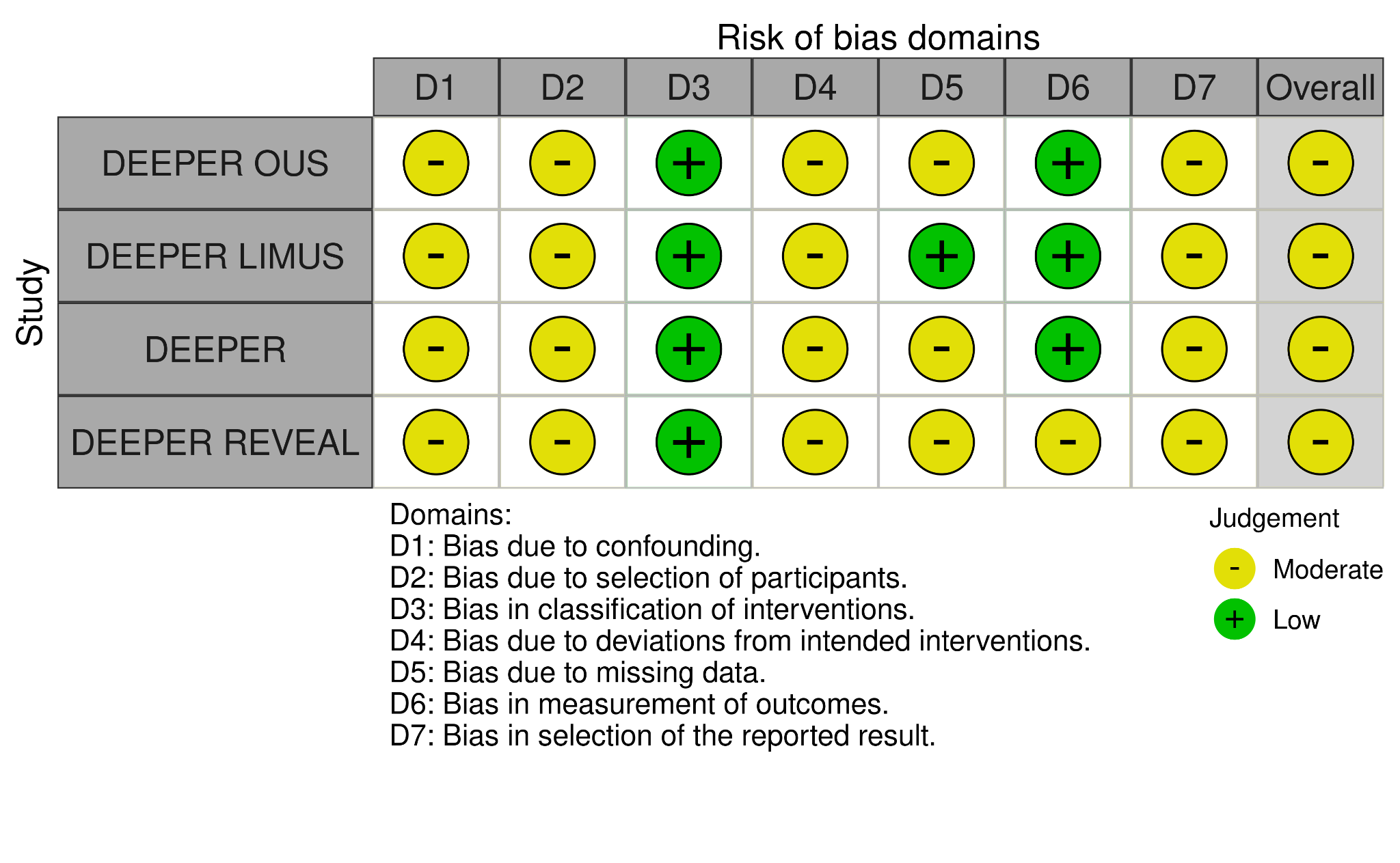


Supplementary Figure 5: Risk of Bias Assessment for non-randomized studies: according to the Cochrane tool ROBINS-I (Traffic light plot)

**PROTOCOL**

This study-level Bayesian network meta-analysis will be conducted in accordance with the Preferred Reporting Items for Systematic Reviews and Meta-Analyses (PRISMA) 2020 guidelines..

A comprehensive search of Ovid MEDLINE, PubMed, Scopus, the Cochrane Central Register of Controlled Trials (CENTRAL), and ClinicalTrials.gov will be performed from inception to November 10, 2025. Search terms will incorporate both Medical Subject Headings (MeSH) and keywords related to infrapopliteal arterial disease, tibial artery revascularization, percutaneous transluminal angioplasty, drug-coated balloons, drug-eluting stents, bare-metal stents, atherectomy, and retrievable scaffold therapy. No date restrictions will be applied, and bibliographies of eligible studies and relevant reviews will be screened to identify additional publications. Only English language studies will be included.

Eligible studies will be clinical trials enrolling adults with infrapopliteal arterial disease undergoing endovascular revascularization. Studies will be required to report arm-level data for at least one of the prespecified outcomes:

- Primary outcome: Major Adverse Events (MAE) at 30 days
- Secondary outcomes: all-cause mortality at 12 months, clinically-driven target lesion revascularization (CD-TLR) at 6 months, and major limb amputation at 6 months

Comparator categories of interest will include:

- Percutaneous transluminal angioplasty (PTA)
- Angioplasty with drug-coated balloon (DCB)
- Balloon angioplasty followed by drug-eluting stent (DES)
- Balloon angioplasty followed by bare-metal stent (BMS)
- Balloon angioplasty followed by atherectomy
- Balloon angioplasty combined with retrievable scaffold therapy (RST)

Because available RST studies are limited to single-arm designs, these will be retained if they report extractable binomial outcome data; the Bayesian arm-based modeling framework will permit their inclusion by allowing single-arm evidence to contribute to baseline risk estimation.

Studies will be excluded if they enroll patients with mixed lesion locations without separate infrapopliteal data, lack extractable event counts or sample sizes, or do not report any primary or secondary outcomes. Two reviewers will independently screen all titles and abstracts, followed by a full-text review. Discrepancies will be resolved by consensus or by consultation with a third reviewer.

Data extraction will be performed in duplicate using a standardized Excel sheet capturing study characteristics, patient demographics, lesion length, comorbidities, treatment assignment, and event counts. Extracted covariates will include mean age, prevalence of diabetes mellitus, smoking prevalence, prevalence of chronic kidney disease, and lesion length. Risk of bias will be evaluated independently by two reviewers using the Cochrane Risk of Bias 2.0 tool.

A Bayesian arm-based network meta-analysis will be conducted using JAGS through R. Each study arm will be modeled using a binomial likelihood with a logit link, decomposing the arm-level log-odds into a study-specific baseline effect and a treatment-specific relative effect. PTA will serve as the reference treatment. Random effects will be applied to account for between-study heterogeneity, and weakly informative priors will be assigned to variance parameters. Convergence will be confirmed by trace plots and the Gelman–Rubin statistic.

Odds ratios with 95% credible intervals will be derived from posterior samples for all pairwise treatment comparisons. Network geometry will be visualized with node-link diagrams, with node sizes reflecting the number of contributing studies and edges weighted by the number of direct comparisons. Treatment rankings will be summarized using SUCRA values.

Publication bias will be evaluated using funnel plots and Egger regression adapted for arm-based data. Sensitivity analyses will be conducted by repeating the network meta-analysis after excluding studies identified as high-risk for publication bias.

Study-level Bayesian meta-regression analyses will be performed for each prespecified covariate (mean age, diabetes mellitus prevalence, smoking prevalence, chronic kidney disease prevalence, and lesion length) individually. Each meta-regression will be restricted to studies reporting that particular covariate. All analyses will be conducted using R version 4.5.1.

## 🔹 PICO Framework

| Element | Description |
| --- | --- |
| Population (P) | Adults with infrapopliteal arterial disease undergoing endovascular revascularization |
| Intervention (I) | Endovascular revascularization with drug-coated balloon (DCB), drug-eluting stent (DES), bare-metal stent (BMS), atherectomy, or retrievable scaffold therapy (RST) |
| Comparator (C) | Percutaneous transluminal angioplasty (PTA) |
| Outcomes (O) | - Primary: Major Adverse Events (MAE) at 30 days - Secondary: all-cause mortality at 12 months, clinically-driven target lesion revascularization (CD-TLR) at 6 months, major limb amputation at 6 months |

## 🔹 Detailed Search Strategy

### **MEDLINE (Ovid)**

Code

1. Peripheral Arterial Disease/ OR Arterial Occlusive Diseases/ OR Tibial Arteries/ OR Popliteal Artery/

2. (infrapopliteal OR tibial OR below-the-knee OR BTK).ti,ab.

3. Angioplasty, Balloon/ OR Angioplasty/ OR Percutaneous Transluminal Angioplasty/

4. (angioplasty OR PTA OR percutaneous transluminal angioplasty).ti,ab.

5. Drug-Coated Balloons/ OR (drug-coated balloon OR DCB).ti,ab.

6. Stents/ OR Drug-Eluting Stents/ OR Bare-Metal Stents/

7. (drug-eluting stent OR DES OR bare-metal stent OR BMS).ti,ab.

8. Atherectomy/ OR (atherectomy).ti,ab.

9. (retrievable scaffold OR bioresorbable scaffold OR RST).ti,ab.

10. Randomized Controlled Trial.pt. OR Clinical Trial.pt. OR controlled clinical trial.pt.

11. 1 OR 2

12. 3 OR 4 OR 5 OR 6 OR 7 OR 8 OR 9

13. 11 AND 12

14. Limit 13 to English language

### **PubMed:**

Code

(("Peripheral Arterial Disease"[MeSH] OR "Arterial Occlusive Diseases"[MeSH] OR "Tibial Arteries"[MeSH] OR infrapopliteal[tiab] OR tibial[tiab] OR "below-the-knee"[tiab])

AND

("Angioplasty, Balloon"[MeSH] OR angioplasty[tiab] OR PTA[tiab] OR "Percutaneous Transluminal Angioplasty"[tiab] OR "Drug-Coated Balloons"[MeSH] OR DCB[tiab] OR "Stents"[MeSH] OR "Drug-Eluting Stents"[MeSH] OR DES[tiab] OR "Bare-Metal Stents"[tiab] OR BMS[tiab] OR "Atherectomy"[MeSH] OR atherectomy[tiab] OR "retrievable scaffold"[tiab] OR "bioresorbable scaffold"[tiab] OR RST[tiab]))

AND (randomized controlled trial[pt] OR clinical trial[pt])

AND English[lang]

### **CENTRAL / Scopus**

- Use similar combinations of MeSH-equivalent subject headings and keywords.
- Boolean operators: (Population terms) AND (Intervention terms) AND (Study design terms)
- Filters: English language, clinical trials

### **ClinicalTrials.gov**

- Search terms: "infrapopliteal" OR "tibial artery" OR "below-the-knee"
- Intervention filters: "angioplasty" OR "drug-coated balloon" OR "drug-eluting stent" OR "bare-metal stent" OR "atherectomy" OR "retrievable scaffold"
- Status: Completed or ongoing trials
